# Altered microbial cargo in fecal microbiome-derived outer membrane vesicles as novel biomarkers for vascular dementia

**DOI:** 10.1186/s12866-026-05040-5

**Published:** 2026-04-21

**Authors:** Xin Li, Wei Wei, Shouchao Wei, Wenwei Xu, Lang Mo, Junjun Wang, He Zhu, Zhou Liu, Fengri Jin

**Affiliations:** 1 Zhanjiang Institute of Clinical Medicine, Central People’s Hospital of Zhanjiang, Yuanzhu Road, Zhanjiang, Guangdong China; 2 Medical Center, Gaomi People’s Hospital, Zhenfu Street, Weifang, Shandong China; 3 The Third Department of Neurology, Central People’s Hospital of Zhanjiang, Yuanzhu Road, Zhanjiang, Guangdong China; 4 Office of Drug Clinical Trial Institution, Central People’s Hospital of Zhanjiang, Yuanzhu Road, Zhanjiang, Guangdong China; 5 Department of Physiology, School of Basic Medical Sciences, Guangdong Medical University, Renmindadao Street, Zhanjiang, Guangdong China; 6 Institute of Neurology, The Affiliated Hospital of Guangdong Medical University, Zhanjiang, Guangdong China

**Keywords:** Vascular dementia, Outer membrane vesicles, Fecal microbiome, High-throughput sequencing, Machine learning

## Abstract

**Background:**

This study aims to analyze the composition, diversity, and metabolic functions of fecal microbiome (FM)-derived outer membrane vesicles (OMVs) in patients with vascular dementia (VaD), to identify potential biomarkers for VaD diagnosis.

**Methods:**

FM-derived OMVs were isolated from 29 VaD patients and 28 matched controls via ultracentrifugation and characterized using transmission electron microscopy and nanoparticle tracking analysis. PKH26-labeled OMVs were used for in vivo tracking in mouse brains. Microbial composition was profiled by 16 S rRNA sequencing, combined with diversity analysis and machine learning.

**Results:**

VaD-OMVs were widely distributed in multiple cognitive function-related regions of mouse brains. The VaD group showed a decreased Chao1 index and increased coverage. β-diversity (PCoA/PLS-DA) revealed significant structural differences. Conditional pathogens (e.g., *Pseudomonas*, *Acinetobacter*) were enriched, while beneficial bacteria (e.g., *Bifidobacterium*) were reduced. Correlation analysis indicated promoting effects of Pseudomonadaceae and inhibitory effects of Faecalibacterium. Metabolic pathways including amino acid, carbohydrate, and nucleotide metabolism were enriched. A random forest model achieved an AUC of 0.74 (95% CI: 0.59–0.88) in classifying VaD.

**Conclusion:**

VaD is associated with distinct OMV microbial and functional profiles. OMV-based biomarkers show potential for VaD diagnosis.

**Supplementary Information:**

The online version contains supplementary material available at 10.1186/s12866-026-05040-5.

## Introduction

Vascular dementia (VaD) is an acquired cognitive impairment syndrome resulting from cerebrovascular pathologies and is characterized by progressive cognitive decline and significant functional deterioration due to cerebral tissue damage [1]. Accounting for 15%–30% of dementia cases worldwide, VaD is the second most common type after Alzheimer’s disease [2, 3]. Its prevalence in developing Asian countries, such as China, reaches 30%, markedly higher than that in Western regions [4], suggesting significant influences from environmental and lifestyle factors. Established vascular risk factors, including hypertension, hyperlipidemia, and type 2 diabetes, are strongly associated with VaD pathogenesis [5–7]. Nonetheless, the exact mechanisms remain unclear, impeding early diagnosis and effective treatment.

A growing body of evidence indicates that the microbiota can mediate central nervous system functions through multiple pathways, providing a new dimension for in-depth exploration of neurological disease mechanisms [8–11]. As the largest symbiotic microbial ecosystem in the human body, the gut microbiota has a biomass of up to 10^14^ [12]. These microorganisms perform vital physiological functions through mechanisms such as immune regulation, maintenance of intestinal barrier integrity, and participation in nutrient metabolism, resulting in their designation as the host’s “second genome” [13, 14]. Several investigations have revealed characteristic alterations in the fecal microbiome (FM) of VaD patients, indicating a potential link between gut microbial dysbiosis and disease pathology [15–18]. Recent studies have also confirmed that fecal samples can adequately reflect the composition of the colonic luminal microbiota, providing a practical basis for non-invasive investigations into the gut-brain axis [19, 20]. Nevertheless, the specific mechanisms through which the microbiome influences VaD onset and progression remain to be fully elucidated.

Outer membrane vesicles (OMVs) are increasingly recognized as novel mediators of bacterium‒host interactions. These nanosized membrane structures (20–350 nm in diameter), which are actively secreted by gram-negative bacteria, play a significant role in the pathogenesis of cerebrovascular diseases [18]. OMVs can activate immune responses and promote the release of inflammatory factors, thereby disrupting blood‒brain barrier integrity and increasing permeability, which facilitates the entry of pathological substances into the brain parenchyma [21–23]. Notably, the RNA, proteins, and lipids carried by OMVs may modulate neuronal and vascular endothelial functions, potentially amplifying the extent of damage or accelerating disease progression [24–28].

**Fig. 1:**
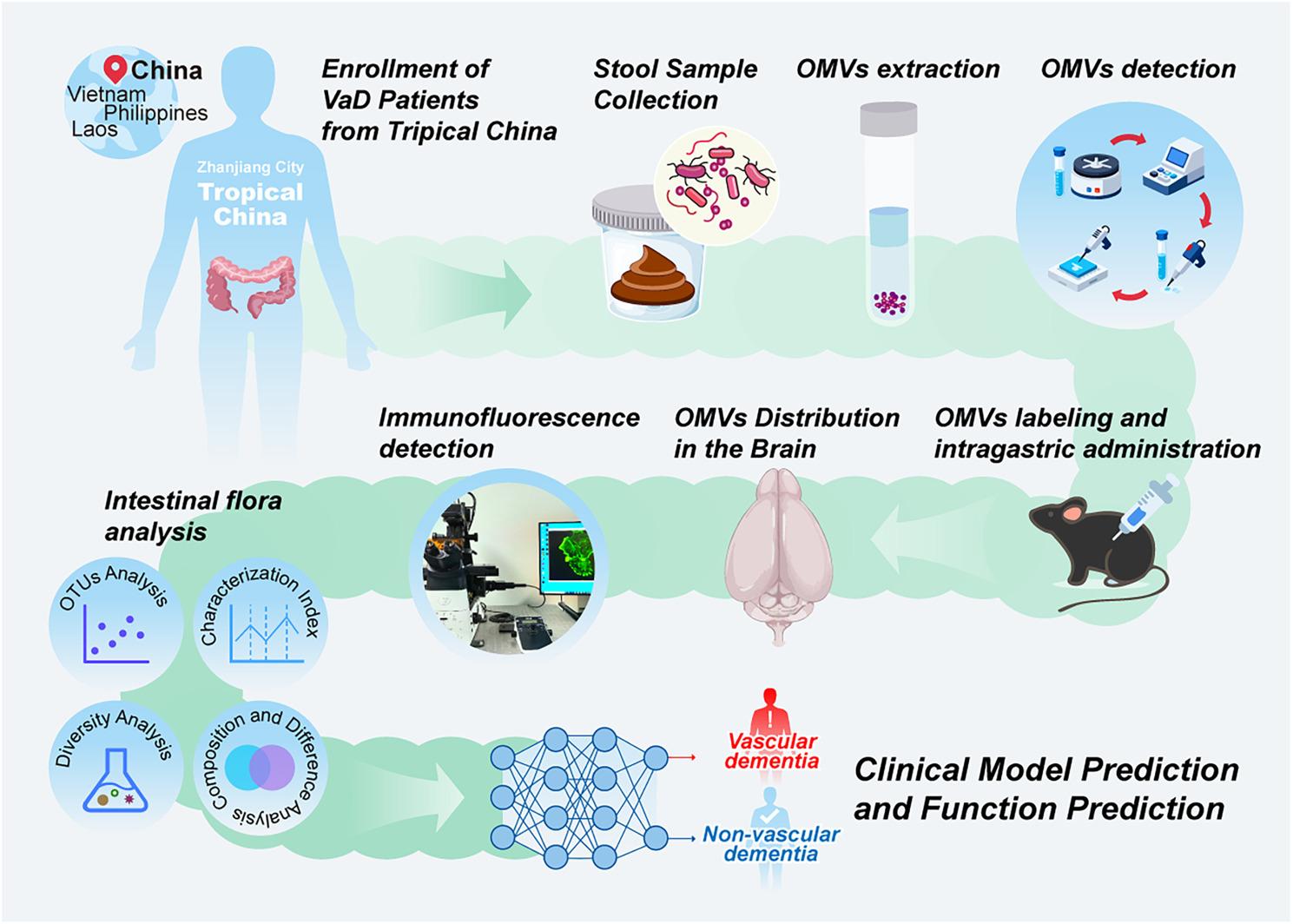
Overall flowchart of this study

While current research has identified molecular and microbial distinctions between VaD patients and healthy controls based on fecal microbiome analysis, the multi-omics features and functional roles of OMVs derived from this specific niche remain largely unexplored. Elucidating the composition and function of intestinal OMVs is critical for understanding VaD pathogenesis, yet this field remains under-investigated. Here, we apply an integrated approach, including 16 S rRNA sequencing of fecal OMV-enriched samples, machine learning, and functional prediction, to systematically profile the OMV-associated microbiome, metabolic activity, and diagnostic utility (Fig. 1). Our work aims to gain insight into host-microbe interactions in VaD and support the development of OMV-based diagnostics and microbiome-targeted therapies.

## Materials and methods

### Patient selection and sample collection

This study was conducted at Central People’s Hospital of Zhanjiang (CPHZ) from February 2024 to July 2025. Eligible VaD patients and age-/sex-matched controls were recruited on the basis of predefined inclusion and exclusion criteria. The clinical assessment was conducted using a structured questionnaire developed for this study (see Clinical and Cognitive Assessment Questionnaire for Vascular Dementia Study). All participants provided qualified fecal samples and completed questionnaire surveys. This study was conducted in accordance with the principles of the Declaration of Helsinki. All procedures were approved by the Institutional Review Board of CPHZ Ethics Committee (Approval No. IIT-2024046-01). Written informed consent was obtained from all participants or their legally authorized representatives prior to enrollment. For details, please refer to the Supplementary Materials. Fresh morning stool samples (≥ 200 g) were collected in sterile containers, immediately sealed in anaerobic bags, and stored at − 80 °C to preserve microbial integrity.

### OMV purification and characterization

On the basis of the literature reports and previous research methods from our research group, we further optimized the extraction and purification procedures of OMVs, with the specific steps as follows [29]: thawed fecal samples were homogenized in ice-cold 0.9% saline via low-frequency pulse homogenization (3–5 s intervals); sequential filtration (70 μm nylon mesh) and differential centrifugation (4 °C) were performed as follows: 500 ×g, 10 min (6 cycles); 1,000 ×g, 15 min (5 cycles); 3,000 ×g, 30 min (3 cycles); 5,000 ×g, 60 min (2 cycles); and the supernatants were filtered (0.45 μm, 0.22 μm, 3 cycles) and ultracentrifuged (100,000 ×g, 2 h, SW32Ti/SW41Ti rotors). OMVs (10 µL) were adsorbed onto ethanol-cleaned copper grids (25 °C, RH 45%, 15 min), negatively stained with 2% phosphotungstic acid (pH 6.8, 5 min), and imaged under 120 kV acceleration (Hitachi HT7800). The size distribution of the OMVs was quantified via a Malvern NS300 (532 nm laser, sCMOS camera, NTA 3.4 software) at 25 °C.

### 16S rRNA sequencing

The purified OMV samples were stored at − 80 °C. The genomic DNA of the microbiome from the fecal samples was extracted via the E.Z.N.A.^®^ Soil DNA Kit (Omega Biotek, USA). The V3-V4 region of the bacterial 16 S rRNA gene was subsequently amplified via the ABI GeneAmp^®^ 9700 PCR Thermal Cycler (ABI, California, USA) with the primers 338 F (5’-ACTCCTACGGGAGGCAGCAG-3’) and 806R (5’-GGACTACHVGGGTWTCTAAT-3’). Following the standard protocol established by Meiji Biomedical Technology Co., Ltd. (Shanghai, China), equimolar purified amplicon pools were prepared for paired-end sequencing on the Illumina MiSeq PE300 platform/NovaSeq PE250 platform (Illumina, San Diego, USA), and raw 16 S rRNA gene sequencing reads were demultiplexed.

### Analysis of 16 S rRNA sequencing data

Operational taxonomic units (OTUs) were clustered via the UPARSE algorithm (version 7.1; http://drive5.com/uparse/) with a 97% similarity cutoff. Taxonomic classification of representative sequences from each OTU was performed via the RDP classifier (version 2.2) on the basis of the 16 S rRNA database (Release 138; http://www.arb-silva.de). Rank-abundance curves were constructed by sorting the OTUs in descending order on the basis of sequence count and plotted via R language tools. Pan/core species analysis was conducted at the taxonomic level of the OTUs, with analytical visualization performed via the vegan package (version 2.4.3) in R (version 3.3.1). α-Diversity analysis, which is based on the Chao1, Sobs, Shannon, ACE, and coverage indices, was employed to assess the species richness and structural heterogeneity of the microbial communities derived from OMVs. β-Diversity analyses, including principal coordinate analysis (PCoA) and partial least squares discriminant analysis (PLS-DA), were performed. The Gut Microbiome Health Index (GMHI) was used to evaluate host health status.

At the genus level, we identified the core community of VaD-derived bacteria and compared the environmental sensitivity of the OMV-associated microbial communities. This comparison included the mean relative abundance (abundance) and the detection frequency (number) of three persistence types across samples: transient, intermediate, and persistent taxa [30, 31]. A linear regression analysis (with reported *R²* values and *P* values) was performed to correlate the species detection frequency with the mean relative abundance across all samples. Furthermore, we employed keystone species analysis to screen the top 10 taxa ranked by the median of the structural keystone index. This approach effectively identified the keystone species within the microbial community [32].

The bacterial community composition of each sample was quantified at the phylum, class, order, family, and genus levels. To visually represent unique and shared operational taxonomic units (OTUs) present across multiple samples, Venn diagrams were generated to calculate the number of species present in each sample. On the basis of parametric/nonparametric testing strategies, intergroup significance difference tests were employed to resolve structural heterogeneity in the microbiome between groups. We also utilized linear discriminant analysis effect size (LEfSe) to perform hierarchical differential analysis of microbiome data through biomarker screening across taxonomic hierarchies (phylum to species). Linear discriminant analysis (LDA) was applied to estimate the effect size of each component (species) on differential effects.

This study employed random forest analysis, an ensemble learning model, to jointly interpret the multidimensional feature spaces of samples by constructing multidecision tree classifiers. The receiver operating characteristic (ROC) curve was used to reveal the trade-off between sensitivity and specificity through dynamic threshold adjustments. Single-factor correlation networks were constructed to analyze species‒species correlations, with network attributes enabling the identification of key species implicated in disease progression. PICRUSt2 was utilized as a microbiome functional prediction tool to infer the functional composition of microbial communities. The distributions of distinct bacterial taxa identified in sample populations were visualized via R software (version 3.3.1) and the mixOmics package. Analyses were performed via R (version 3.3.1), the ade4 package, and the cluster package. Data processing was conducted on the Majorbio Cloud Platform (www.majorbio.com).

### Animals

The experimental animals used in this study were two-week-old male C57BL/6J mice (specific pathogen-free [SPF] grade, body weight 20 ± 3 g) provided by Hangzhou Ziyuan Biotechnology Co., Ltd. (License No. SCXK [Zhe] 2019-0004). Adaptive housing was conducted at the SPF-level animal experimental center of Guangdong Medical University.

### Anesthesia method

To ensure animals were in a state of deep analgesia and unconsciousness prior to brain tissue perfusion and collection, thereby eliminating pain and stress during surgical procedures. Experimental animals were all subjected to deep anesthesia prior to euthanasia, ensuring they remained completely unconscious throughout the critical operative steps. 1.25% tribromoethanol (Avertin) solution (M2920, Nanjing AlBi Bio-Technology Co.Ltd, China) was used. This agent is a commonly used short-acting anesthetic characterized by rapid onset, moderate duration of anesthesia, and stable anesthetic depth, making it suitable for acute surgical procedures in rodent models. M2920 is a ready-to-use sterile solution containing 1.25% (v/v) tribromoethanol (Avertin), tertiary amyl alcohol, and 0.9% physiological saline, with a final tribromoethanol concentration of 20 mg/ml. Administration was via intraperitoneal injection at a dose of 0.2 ml/10 g body weight for mice. This dosage ensures that animals enter a stable surgical plane of anesthesia within minutes, as indicated by the loss of corneal and toe-pinch reflexes.

### Euthanasia/sacrifice method

Following deep anesthesia (unconsciousness), transcardiac perfusion fixation was employed as the terminal procedure. This method enables both humane euthanasia and optimal preservation of tissue architecture for high-quality neurohistological analysis. Detailed Procedure: the deeply anesthetized mouse was positioned supine and secured on a surgical board; the thoracic cavity was quickly opened to expose the heart; a perfusion needle was inserted into the ascending aorta via the left ventricle, and the right atrial appendage was incised to serve as an outflow tract; approximately 20 mL of ice-cold 0.9% physiological saline was rapidly perfused first, until the liver and lungs turned pale and the effluent from the right atrial appendage ran clear, ensuring thorough removal of blood from the vasculature; this was followed by perfusion with approximately 50 mL of ice-cold 4% paraformaldehyde (PFA) in phosphate buffer, continuing until rigidity and tremors were observed in the limbs, tail, and torso of the animal, after which perfusion was stopped.

All animal experimental protocols and procedures were reviewed and approved by the Institutional Animal Ethics Committee of Guangdong Medical University (Ethics Approval No. GDY2403466). For details, please refer to the Supplementary Materials.

### Fluorescent labeling of OMVs

To clarify the distribution of VaD-OMVs in the brains of C57BL/6J mice, PKH67 fluorescent dye was used to label VaD-OMVs. OMVs were incubated with PKH67 dye at 4 °C for 2 h. Unbound PKH67 was removed via ultracentrifugation using an SW 60 Ti rotor at 100,000 × g and 4 °C for 1 h. The pelleted material was resuspended in PBS to obtain the labeled product. Fasted C57BL/6J mice (after 24 h of fasting) were orally administered labeled OMVs at a dose of 20 µg/200 µL via oral gavage.

### Mouse brain tissue harvesting and immunofluorescence

The mice were anesthetized via intraperitoneal injection. After complete anesthesia, the brains were harvested and fixed. Brain tissue Sect.  (35 μm thick) were prepared via a cryostat microtome via the sectioning-mounting method. The distribution of VaD-OMVs in various brain regions was observed via a 10x objective lens under an inverted laser confocal microscope (A1-SHR-LFOV, Nikon, Japan).

## Results

### Clinical baseline characteristics of VaD patients and controls

Baseline demographic and clinical characteristics demonstrated no significant intergroup differences in age, sex distribution, or comorbidities (hypertension, diabetes, hyperlipidemia, valvular heart disease, or COPD; all *P* > 0.05; Table 1). However, VaD patients exhibited markedly impaired neurocognitive function compared with controls, as evidenced by lower MMSE (*t* = 17.454, *P* < 0.001) and MoCA (*t* = 20.798, *P* < 0.001) scores and higher HIS scores (*t* = -18.783, *P* < 0.001). These findings align with the diagnostic criteria for vascular cognitive impairment.

**Table 1:** Demographic and clinical comparisons between the VaD and control groups

Variable	VaD Group (*n* = 29)	Control Group (*n* = 28)	Statistical Value	*P* Value
Age (years)	68.59 ± 10.50	67.65 ± 8.78	*t*=-0.818	0.417
Male	13/29	9/28	*χ²*=0.967	0.325
Hypertension	18/29	13/28	*χ²*=1.405	0.236
Diabetes mellitus	6/29	4/28	Fisher’s exact test	0.740
Hyperlipidemia	5/29	5/28	*χ²*=0.004	0.951
Valvular heart disease	8/29	4/28	Fisher’s exact test	0.331
COPD	0/29	1/28	Fisher’s exact test	0.491
MoCA	10.59 ± 4.23	27.46 ± 0.74	*t* = 20.798	< 0.001
MMSE	12.55 ± 4.66	28.50 ± 1.32	*t* = 17.454	< 0.001
HIS	11.66 ± 2.60	1.89 ± 1.03	*t*=-18.783	< 0.001

*Abbreviations*: *VaD* vascular dementia, *Con* control, *COPD* chronic obstructive pulmonary disease, *MMSE* Mini-Mental State Examination, *MoCA* Montreal Cognitive Assessment, *HIS* Hachinski Ischemic Scale

### Morphological characteristics of OMVs

**Fig. 2:**
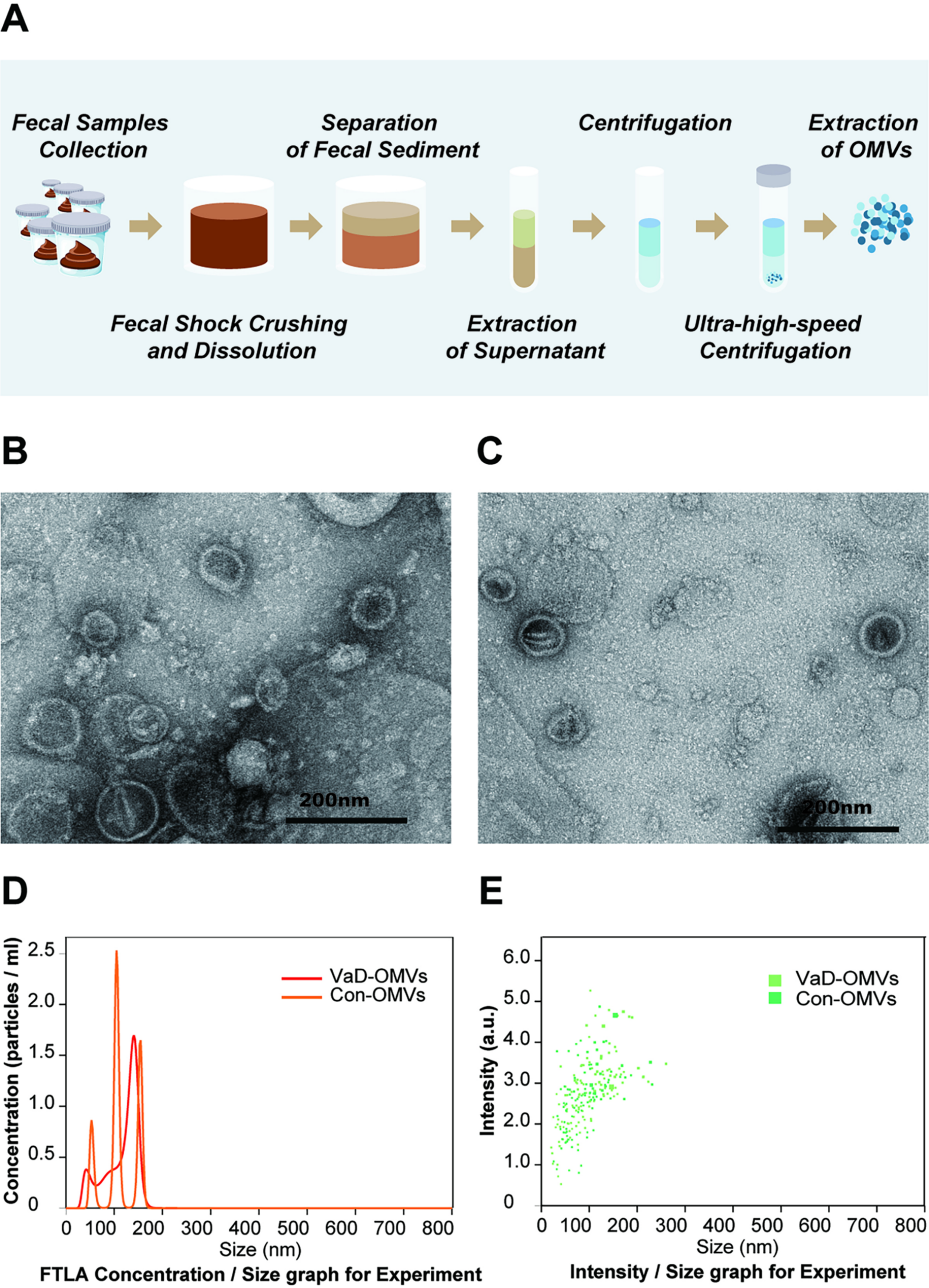
Isolation and characterization of OMVs. **A** Workflow for the extraction and purification of OMVs. **B–C** Representative TEM images of OMVs isolated from VaD patients (**B**) and control subjects (**C**). **D**–**E** NTA of OMVs derived from the VaD and control groups, showing the particle concentration (**D**) and size distribution by intensity (**E**). Abbreviations: OMVs, outer membrane vesicles; VaD, vascular dementia; VaD-OMVs, vascular dementia-derived outer membrane vesicles; Con-OMVs, control-derived outer membrane vesicles; TEM, transmission electron microscopy; NTA, nanoparticle tracking analysis

This study investigated the morphological and dimensional features of OMVs isolated from the FM of individuals with VaD compared with those from control subjects. Visualization by transmission electron microscopy (TEM) revealed that both VaD- and control-derived OMVs displayed smooth-edged, well-defined, and nearly spherical nanostructures (Fig. 2B and C). Particle size analysis indicated that the OMVs from both groups predominantly fell within a size range of 0–200 nm. Consistent with these findings, nanoparticle tracking analysis (NTA) revealed no statistically significant differences in vesicle size distribution between the two groups (Fig. 2D and E).

### Cerebral distribution of VaD-derived OMVs

**Fig. 3:**
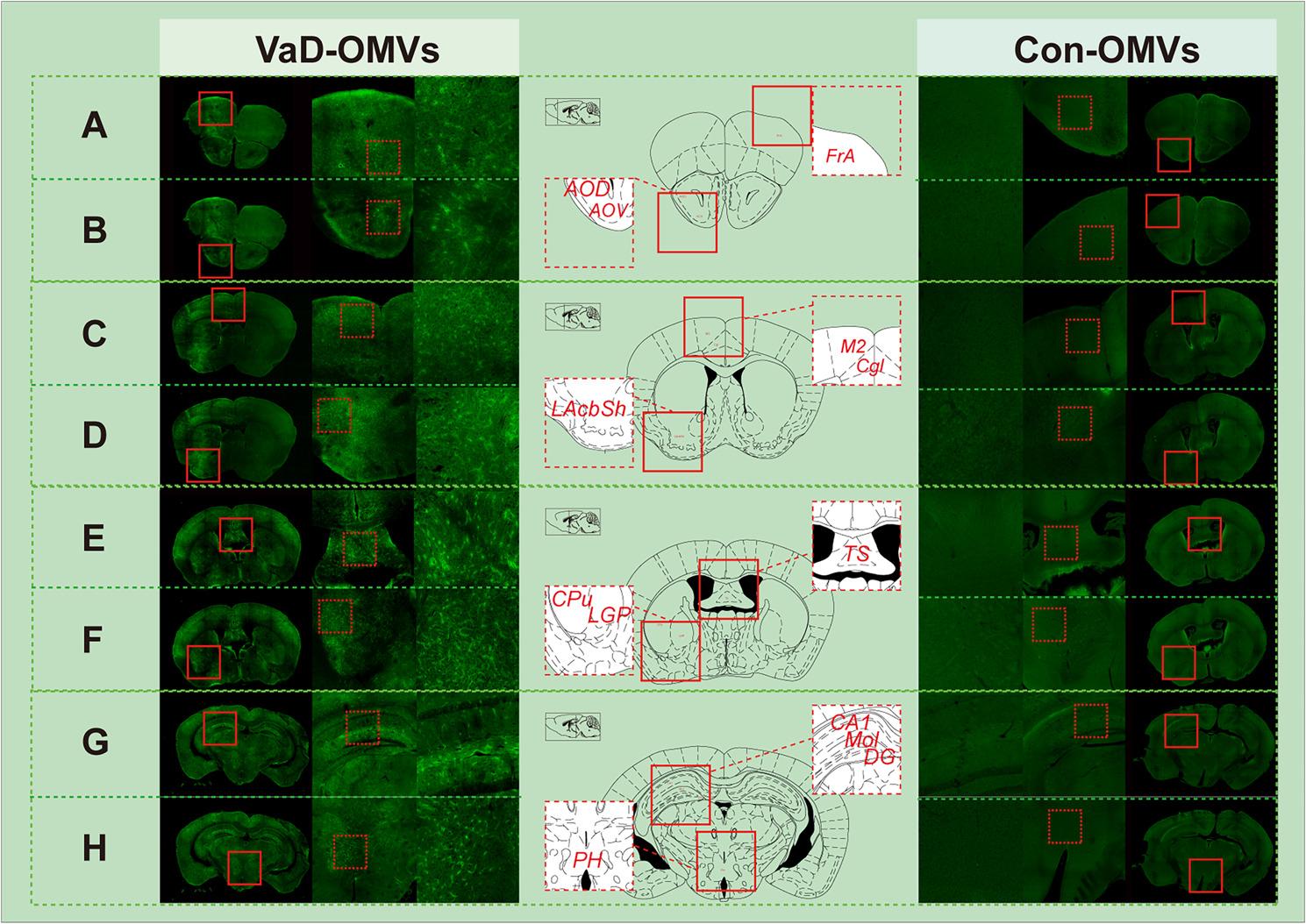
Regional distribution of VaD-OMVs in the mouse brain. **A**–**I** Fluorescence imaging showing the presence of VaD-OMVs in distinct brain regions: (**A**) FrA; (**B**) AOD and AOV; (**C**) M2 and Cgl; (**D**) LAcbSh; (**E**) TS; (**F**) CPu and LGP; (**G**) CA1, Mol and DG; (**H**) PH.Abbreviations: VaD-OMVs, vascular dementia-derived outer membrane vesicles; FrA, frontal association cortex; AOD, anterior olfactory nucleus; AOV, anterior olfactory nucleus, ventral part; M2, secondary motor cortex; Cgl, cingulate cortex, lateral part; LAcbSh, lateral part of the Accumbens Shell; TS, triangular septal nucleus; LGP, lateral globus pallidus; CPu, caudate putamen; Mol, molecular layer of the cerebellum; DG, dentate gyrus; CA1, cornu Ammonis area 1; PH, posterior hypothalamic area

PKH26-labeled OMVs were administered via oral gavage, and their biodistribution in the mouse brain was evaluated via immunofluorescence. Widespread dispersion of OMVs was observed across multiple brain regions implicated in cognitive function, including the frontal association cortex (FrA) (Fig. 3A), the anterior olfactory nuclei (dorsal and ventral parts, AOD and AOV) (Fig. 3B), the secondary motor cortex (M2) and cingulate cortex (Cg) (Fig. 3C), the lateral shell of the nucleus accumbens (LAcbSh) (Fig. 3D), the triangular septum (TS) (Fig. 3E), the caudate putamen (CPu) and lateral globus pallidus (LGP) ( Fig. 3F), the hippocampal CA1 region, molecular layer (Mol), and dentate gyrus (DG; Fig. 3G), as well as the posterior hypothalamus (PH) (Fig. 3H).

### Reduced microbial diversity and significant dysbiosis in VaD-OMV-associated microbiome

**Fig. 4:**
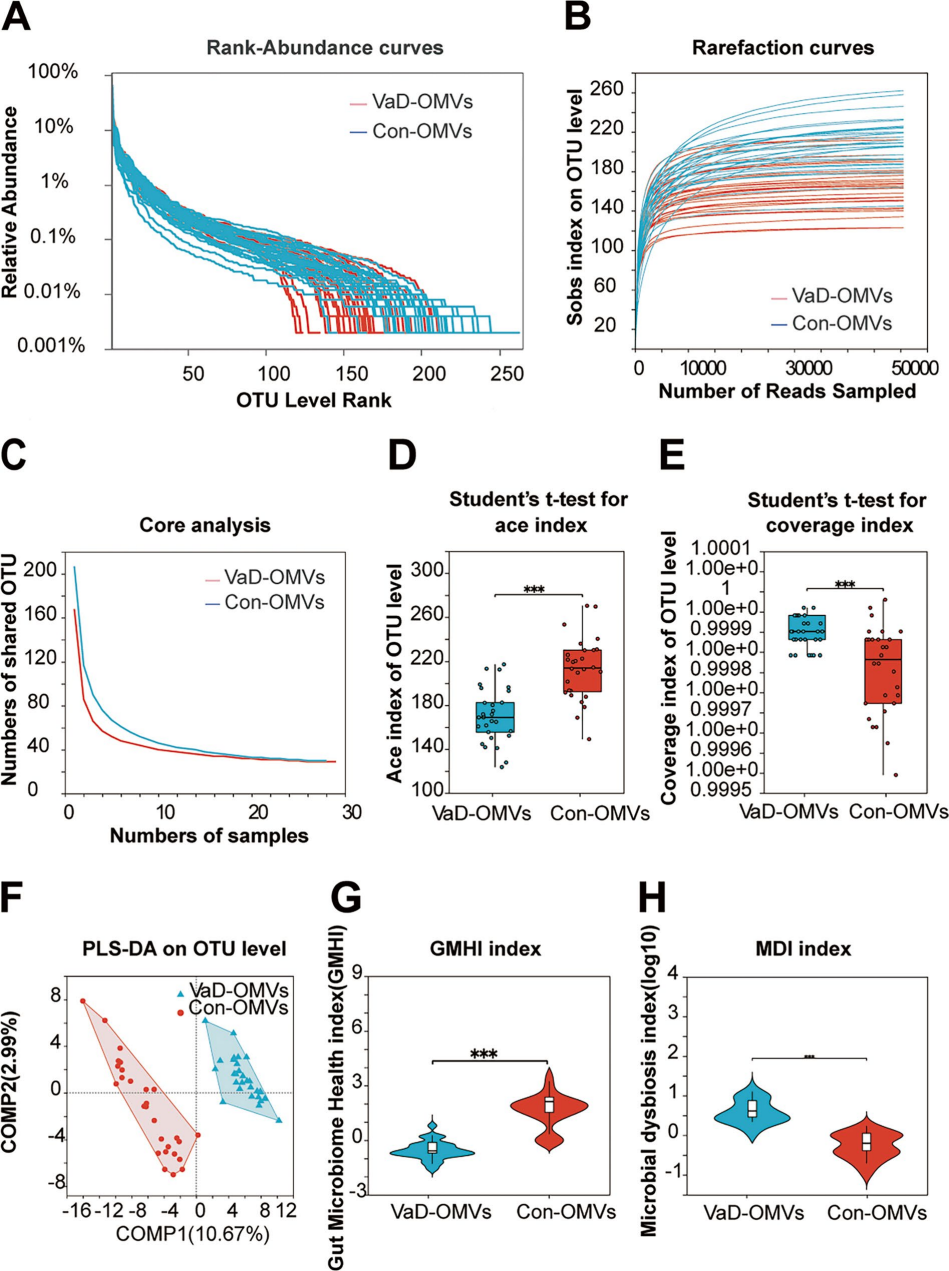
Microbial diversity and dysbiosis analysis of VaD-OMV-associated microbiome. **A** Rank–abundance curves depicting species richness and evenness. **B** Rarefaction curves based on the Sobs index. **C** Core species accumulation curves. **D**-**E** α diversity indices at the OTU level: Ace (**D**) and Coverage (**E**). **F** β diversity analyses: PCoA. **G** Gut microbiome health index GMHI comparison between VaD-OMVs and Con-OMVs groups. **H** MDI comparison between VaD-OMVs and Con-OMVs groups. Abbreviations: VaD-OMVs, vascular dementia-derived outer membrane vesicles; Con-OMVs, control-derived outer membrane vesicles; OTU, operational taxonomic unit; GMHI, Gut Microbiome Health Index; MDI, Microbial Dysbiosis Index; PLS-DA, partial least squares-discriminant analysis

Diversity analysis of 57 samples revealed 4,942,454 high-quality sequences (mean length: 415 bp). Operational taxonomic unit (OTU) clustering and taxonomic alignment were performed to characterize the bacterial composition and abundance. Rank‒abundance curves revealed differences in community structure between VaD-OMVs and Con-OMVs. VaD-OMVs presented greater species abundance but lower evenness (Fig. 4A). Rarefaction analysis based on the Sobs index indicated that sequencing saturation was achieved, with sufficient depth and biological reproducibility (Fig. 4B). Core/Pan species curves confirmed adequate sequencing depth and sample size (Fig. 4C, Supplementary Fig. 1A).

α-Diversity analysis revealed significantly lower bacterial richness in the VaD-OMV group than in the Con-OMV group, as indicated by reductions in the Ace, Chao, and Sobs indices (all *P* < 0.01) (Fig. 4D and Supplementary Fig.s 1B-C). Coverage was greater in VaD-OMVs (*P* < 0.01) (Fig. 4E), whereas the Shannon index was not significantly different (*P* = 0.086) (Supplementary Fig. 1D). β-Diversity analysis revealed clear separation between groups via PCoA (*P* < 0.01) (Supplementary Fig. 1E) and PLS-DA (Fig. 4F), which was supported by significant intergroup dissimilarity (*P* < 0.01) (Supplementary Fig. 1F).

VaD-OMVs also presented a significantly lower gut microbiome health index (GMHI) (*P* < 0.01) (Fig. 4G) and a higher microbial dysbiosis index (MD index) (*P* < 0.01) (Fig. 4H), indicating a compromised microbiome structure compared with those of the controls.

### Microbial community assembly: Prevalence‒abundance relationships and identification of core keystone taxa

**Fig. 5:**
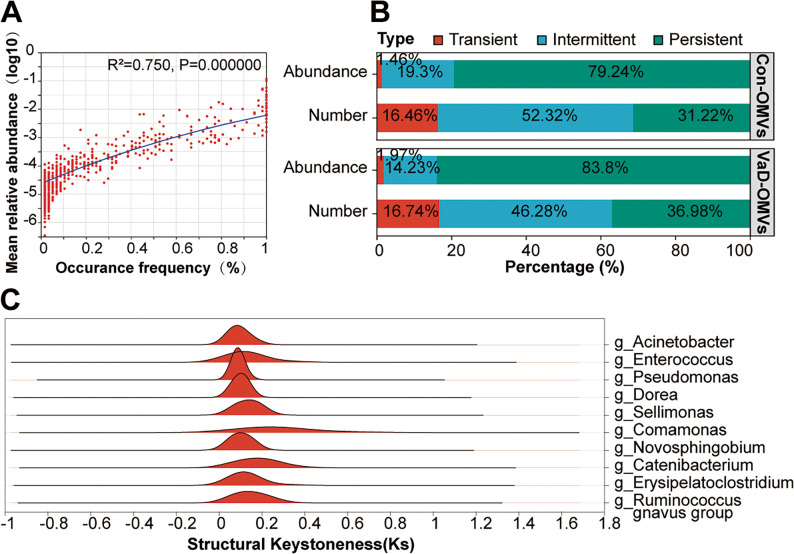
Microbial community assembly: prevalence–abundance relationships and identification of keystone taxa. **A** Correlation between the average abundance and occurrence frequency of ASVs in the OMV-derived microbiome. **B** Number and relative abundance of ASVs across different occurrence frequency categories. **C** Keystone species analysis showing the top 10 taxa ranked by median centrality. Abbreviations: VaD-OMVs, vascular dementia-derived outer membrane vesicles; Con-OMVs, control-derived outer membrane vesicles; ASV, amplicon sequence variant

To elucidate microbial community assembly mechanisms, we assessed the relationship between FM prevalence and mean relative abundance. A strong positive correlation was observed (*R*² = 0.750, *P* < 0.001) (Fig. 5A), indicating that widely distributed species tend to be more abundant.

Microorganisms were classified into persistent, intermediate, and transient taxa on the basis of their prevalence. Although intermediate (46.28%–52.32%) and transient taxa (16.46%–16.74%) contributed more to taxonomic richness, persistent taxa constituted a smaller proportion (31.22%–36.98%) (Fig. 5B, “Number”). In contrast, persistent taxa dominated in terms of relative abundance (79.24%–83.8%), far exceeding intermediate (14.23%–19.3%) and transient taxa (1.46%–1.97%) (Fig. 5B, “Abundance”), suggesting that while diversity is sustained by rare and transitional species, ecosystem function is driven primarily by a persistent core.

We further identified keystone taxa via a structural keystone index (Fig. 5C). The top 10 keystone species included *Acinetobacter* and *Enterococcus*, which presented the highest indices, indicating central roles in the microbial network. Other key taxa, such as *Pseudomonas*, *Ruminococcus gnavus*, and *Dorea*, are also likely critical for maintaining community stability and function.

### Significant shifts in FM taxonomy across multiple levels in VaD-OMVs

**Fig. 6:**
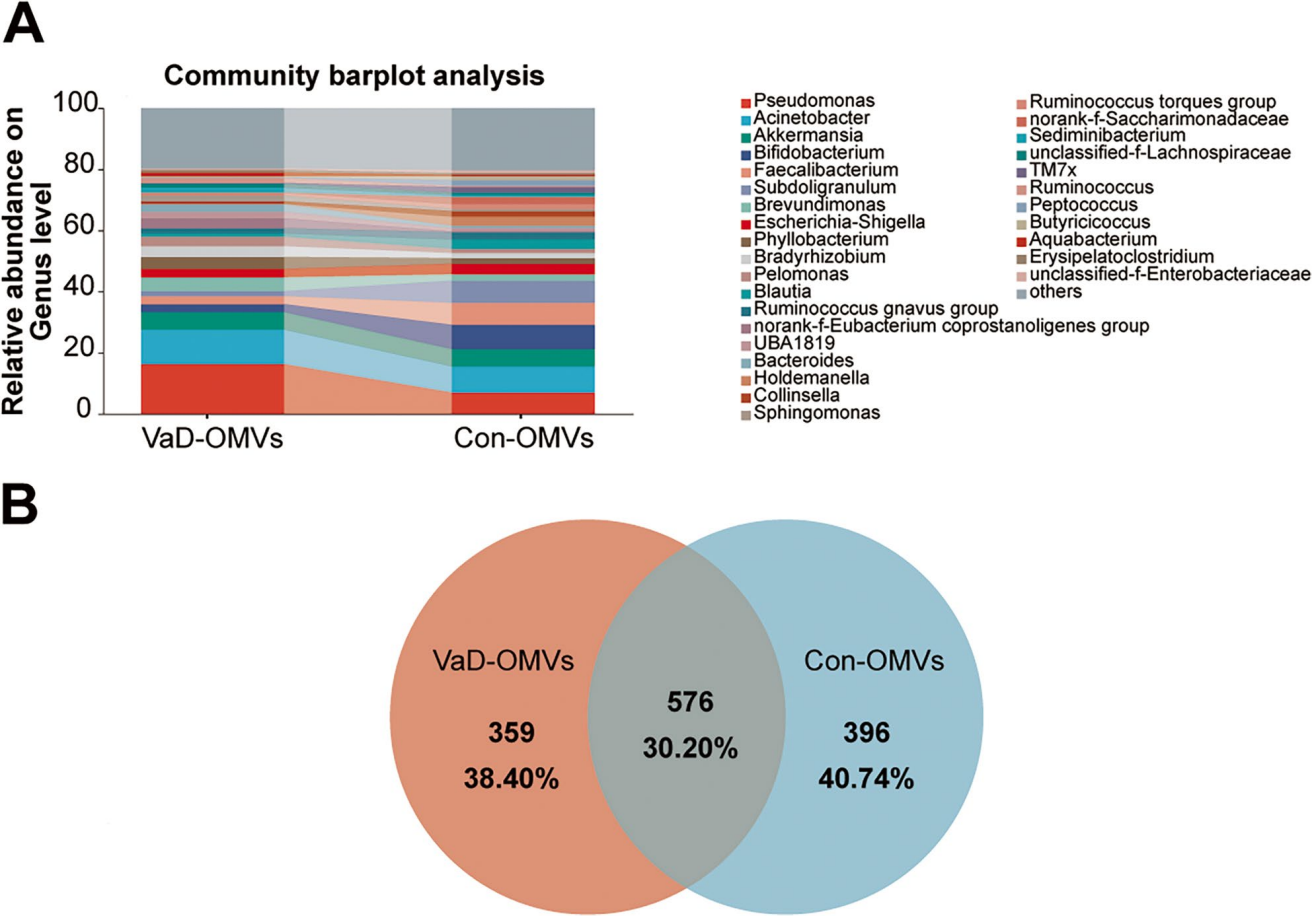
Gut microbial composition of VaD-derived and control-derived OMVs across taxonomic levels. Relative abundance of bacterial communities at the phylum (**A**), class (**B**), order (**C**), family (**D**), and genus (**E**) levels. **F** Venn diagram illustrating shared and unique genera between the two groups. Abbreviations: VaD-OMVs, vascular dementia-derived outer membrane vesicles; Con-OMVs, control-derived outer membrane vesicles

FM composition analysis across taxonomic levels revealed significant shifts in VaD-OMV-associated FM compared with those in controls (Supplementary Fig. 2A-D, Fig. 6A). At the phylum level, VaD-OMVs increased *Proteobacteria* (55.73% vs. 33.18%) and *Bacteroidota* (5.97% vs. 3.41%) but decreased *Firmicutes* (27.22% vs. 41.74%) and *Actinobacteriota* (4.21% vs. 11.26%). Similarly, genera such as *Pseudomonas* (16.34% vs. 7.01%), *Acinetobacter* (11.26% vs. 8.54%), and *Brevundimonas* (4.52% vs. 2.05%) were enriched in VaD-OMVs, whereas *Bifidobacterium* (2.64% vs. 8.00%), *Faecalibacterium* (2.60% vs. 7.17%), and *Subdoligranulum* (1.61% vs. 7.29%) were reduced. Venn analysis revealed 359 unique genera in VaD-OMVs, 396 in controls, and 576 in common (Fig. 6B).

### Significant enrichment of specific FMs in VaD-OMVs

**Fig. 7:**
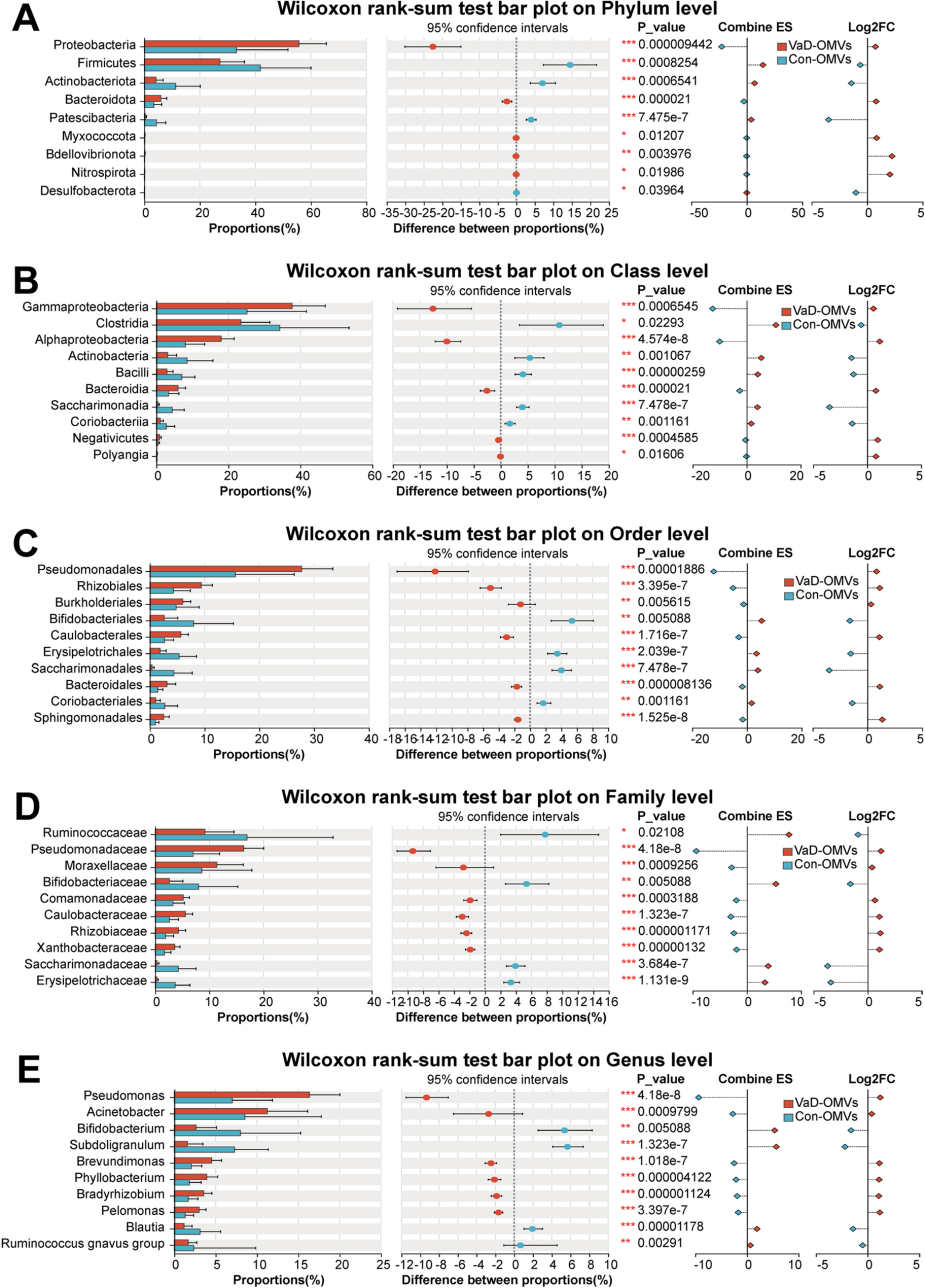
Differential abundance analysis of OMV-derived FM between the VaD and control groups. Significantly altered taxa at the phylum (**A**), class (**B**), order (**C**), family (**D**), and genus (**E**) levels. Statistical methods: Wilcoxon rank-sum test; false discovery rate (FDR) correction; bootstrap confidence interval = 0.95. Abbreviations: VaD-OMVs, vascular dementia-derived outer membrane vesicles; Con-OMVs, control-derived outer membrane vesicles; FM, fecal microbiome

**Fig. 8:**
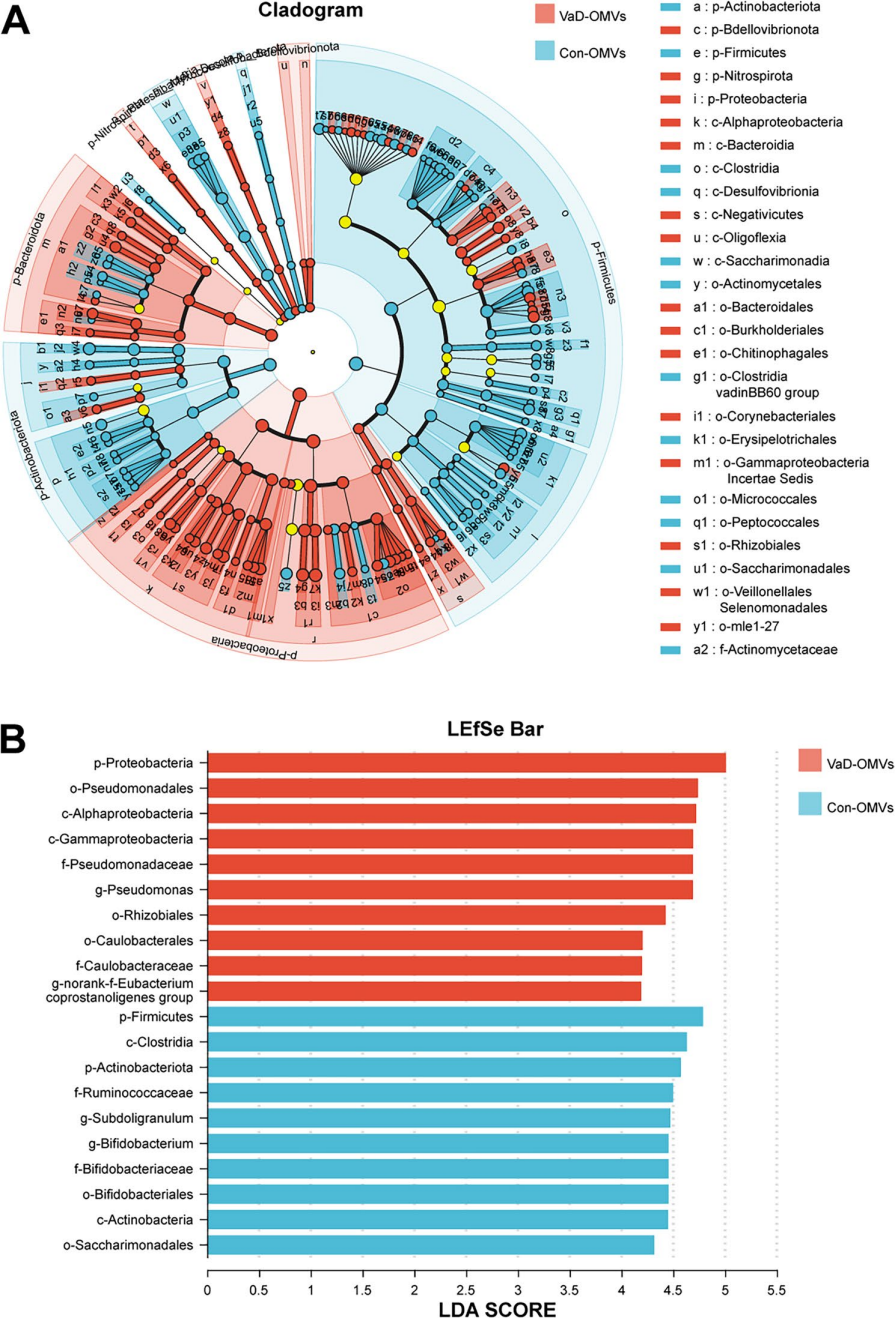
LEfSe analysis identifying differentially abundant taxa across multiple taxonomic ranks. **A** Cladogram generated from LDA effect size (LEfSe) showing enriched taxa in each group. **B** Histogram of LDA scores indicating effect sizes of significantly discriminative taxa. Analysis parameters: all-against-all multigroup comparison; taxonomic levels: phylum to genus. Abbreviations: VaD-OMVs, vascular dementia-derived outer membrane vesicles; Con-OMVs, control-derived outer membrane vesicles; LDA, linear discriminant analysis; LEfSe, linear discriminant analysis effect size

Differential abundance analysis revealed significant taxonomic shifts between the VaD- and Con-OMV groups (Supplementary Tables 1 and Fig. 7A-E). VaD-OMVs presented increased abundances of *Proteobacteria* (55.73 ± 9.83 vs. 33.18 ± 18.57) and *Bacteroidota* (5.97 ± 2.11 vs. 3.41 ± 2.85) at the phylum level (Fig. 7A). At the class level (Fig. 7B), *Gammaproteobacteria* (37.72 ± 9.20 vs. 25.15 ± 16.54), *Alphaproteobacteria* (18.01 ± 3.58 vs. 8.02 ± 5.37), and *Bacteroidia* (5.97 ± 2.11 vs. 3.41 ± 2.85) were significantly enriched. At the order level (Fig. 7C), *Pseudomonadales* (27.74 ± 5.69 vs. 15.60 ± 10.75), *Rhizobiales* (9.37 ± 2.06 vs. 4.30 ± 3.10), *Burkholderiales* (6.02 ± 1.45 vs. 4.80 ± 4.20), and *Caulobacterales* (5.65 ± 1.36 vs. 2.65 ± 1.70) increased. At the family level (Fig. 7D), *Pseudomonadaceae* (16.34 ± 3.69 vs. 7.01 ± 4.88), *Moraxellaceae* (11.40 ± 4.87 vs. 8.60 ± 9.25), and *Caulobacteracea*e (5.59 ± 1.33 vs. 2.63 ± 1.67) were enriched. At the genus level (Fig. 7E), *Pseudomonas* (16.34 ± 3.69 vs. 7.01 ± 4.88), *Acinetobacter* (11.26 ± 4.87 vs. 8.54 ± 9.24), and *Brevundimonas* (4.52 ± 1.19 vs. 2.05 ± 1.27) were significantly enriched in VaD-OMVs (all *P* < 0.05). LEfSe analysis confirmed significant enrichment of *Proteobacteria* and related taxa (e.g., *Pseudomonadales*) in VaD-OMVs, whereas *Firmicutes*, *Actinobacteria*, and *Bifidobacterium* were enriched in Con-OMVs (LDA > 3.5, *P* < 0.05) (Fig. 8A and B), indicating their potential as group-specific biomarkers.

### A random forest model for differentiating VaD based on OMV-derived FM with diagnostic potential

**Fig. 9:**
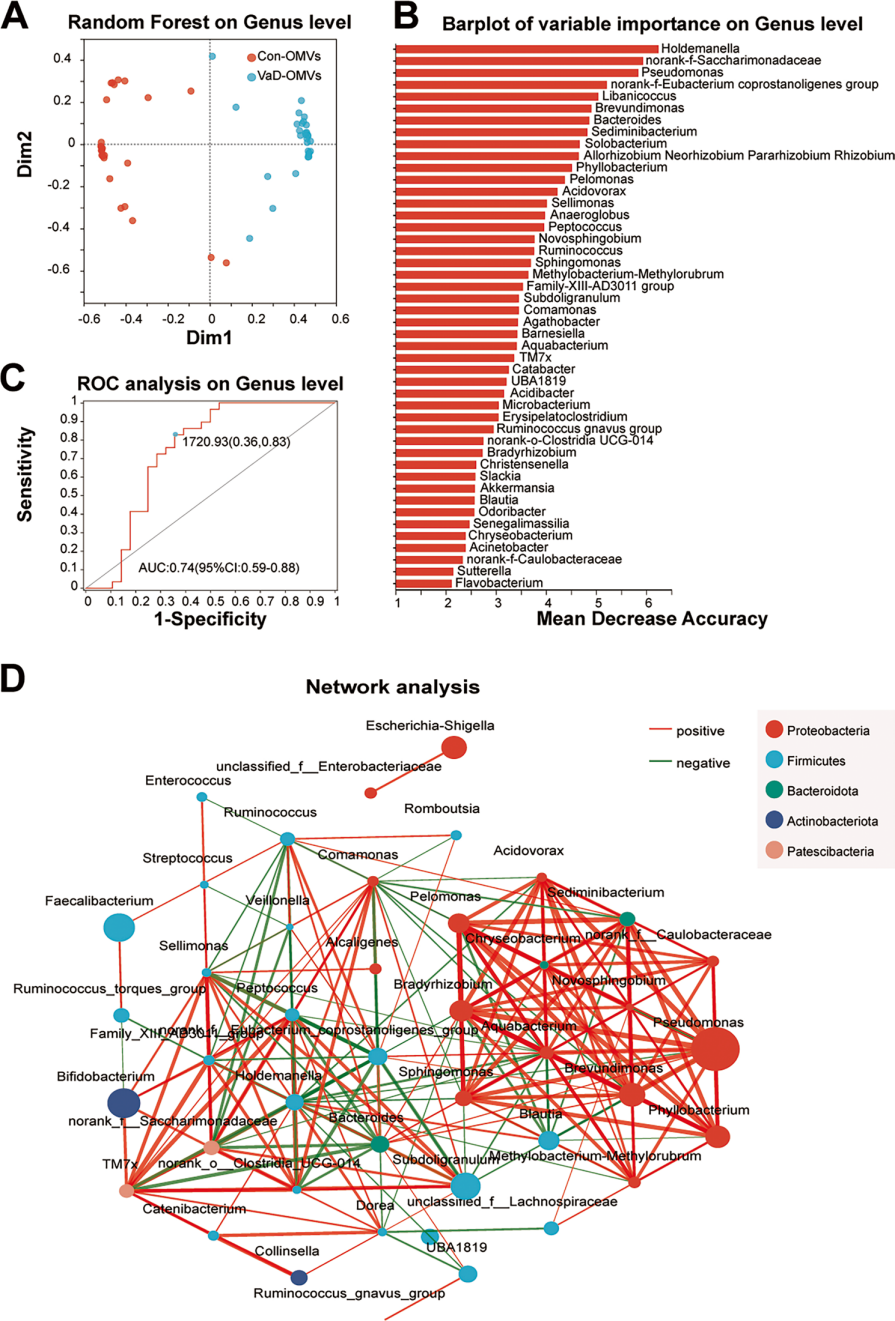
Integrated diagnostic model and functional predictions of the OMV microbiome in VaD. **A** Random forest classification plot based on microbiome composition. **B** The top 10 bacterial features contributing to VaD diagnosis identified by random forest regression. **C** Spearman correlation network of prevalent genera; node color indicates abundance, and edge color represents the correlation coefficient (red: positive, blue: negative). **D** ROC curve analysis of the OMV-derived microbiome at the genus level. Abbreviations: VaD-OMVs, vascular dementia-derived outer membrane vesicles; Con-OMVs, control-derived outer membrane vesicles; ROC, Receiver Operating Characteristic

In this study, a random forest algorithm was applied to construct a machine learning model for assessing the classification performance and taxonomic composition differences between the VaD-OMV and Con-OMV groups. A two-dimensional scatterplot generated from the random forest proximity matrix revealed clear spatial separation between the two groups, reflecting significant intergroup differences in microbial composition (Fig. 9A). To identify the FM taxa derived from the OMVs that contribute most to the diagnosis of VaD-OMVs, the top 10 discriminant genera were selected on the basis of the mean decrease in accuracy (or Gini importance) from the random forest regression (Fig. 9B). At the genus level, the following taxa were ranked by their contribution values: *Holdemanella* (6.24), *norank-f-Saccharimonadaceae* (5.93), *Pseudomonas* (5.84), *norank-f-Eubacterium_coprostanoligenes_group* (5.21), *Libanicoccus* (5.04), *Brevundimonas* (4.90), *Bacteroides* (4.86), *Sediminibacterium* (4.82), *Solobacterium* (4.67), and *Allorhizobium-N-P-R* (4.65).

For diagnostic evaluation, receiver operating characteristic (ROC) analysis revealed an area under the curve (AUC) of 0.74 (95% CI: 0.59–0.88) (Fig. 9C), indicating that the random forest model based on the FM structure can effectively differentiate VaD patients from non-VaD controls and supporting its potential clinical utility.

We constructed a correlation network based on Spearman’s rank coefficients to investigate FM sample relationships through the visualization of abundance correlations across common FM genera. Network analysis revealed decreased complexity in the VaD-OMV group compared with the Con-OMV group (Fig. 9D). Notably, *Pseudomonas* showed positive interactions with multiple taxa, whereas *Faecalibacterium* appeared to exhibit inhibitory relationships.

### Functional prediction of the OMV-derived microbiome in VaD

**Fig. 10:**
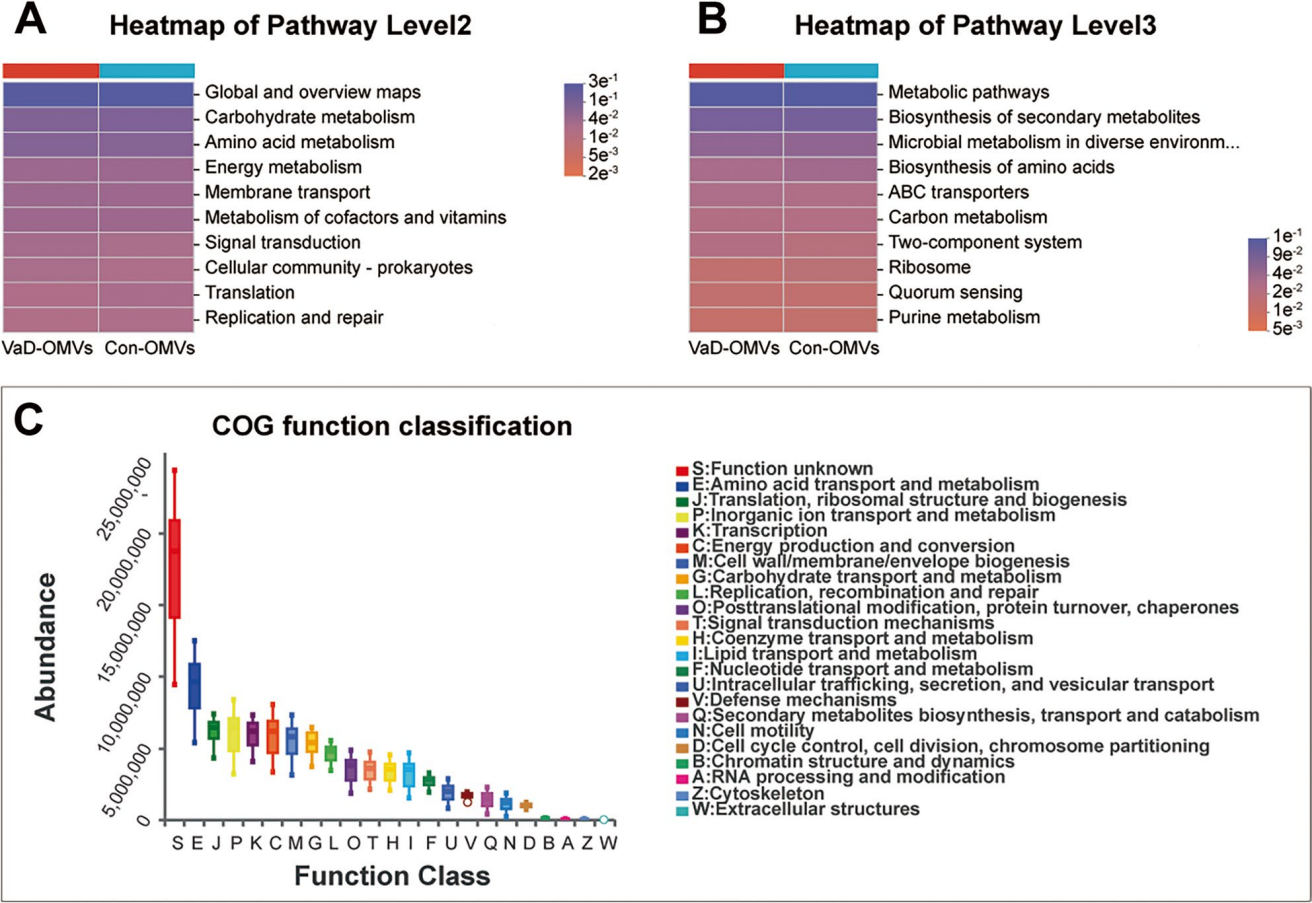
Functional prediction of the OMV-derived microbiome in VaD patients. **A** Functional prediction of the OMV-derived microbiota in VaD patients was performed via PICRUSt2 in conjunction with the KEGG database. **B**-**C** Results of KEGG pathway analysis at level 2 (**B**) and level 3 (**C**). Abbreviations: VaD-OMVs, vascular dementia-derived outer membrane vesicles; Con-OMVs, control-derived outer membrane vesicles; COG, Clusters of Orthologous Groups

In this study, PICRUSt2 was employed to predict the functional profiles of FM-derived OMVs on the basis of 16 S rRNA sequencing data. Through analysis of functional composition and abundance, we investigated the potential mechanistic contributions of OMV-associated bacterial communities to the pathogenesis of VaD. Integrated annotation with the COG database indicated that amino acid metabolism represented the predominant functional category, with its relative abundance significantly surpassing that of other metabolic modules (Fig. 10A).

Further functional prediction via the KEGG database revealed that, at the primary functional level, the microbiome was predominantly enriched in carbon and nitrogen metabolic networks, encompassing pathways involved in carbohydrate, amino acid, and nucleotide metabolism (Fig. 10B).

At the tertiary KEGG level, OMV-associated functions clustered into three major modules: (1) metabolic regulation, including secondary metabolite biosynthesis, amino acid biosynthesis, and purine metabolism; (2) environmental adaptation mechanisms, such as ABC transporters, two-component systems, and quorum sensing; and (3) fundamental cellular functions, exemplified by ribosome assembly and central carbon metabolic pathways (Fig. 10C).

### Analysis of OMVs reveals key microbial taxa associated with clinical factors in VaD

**Fig. 11:**
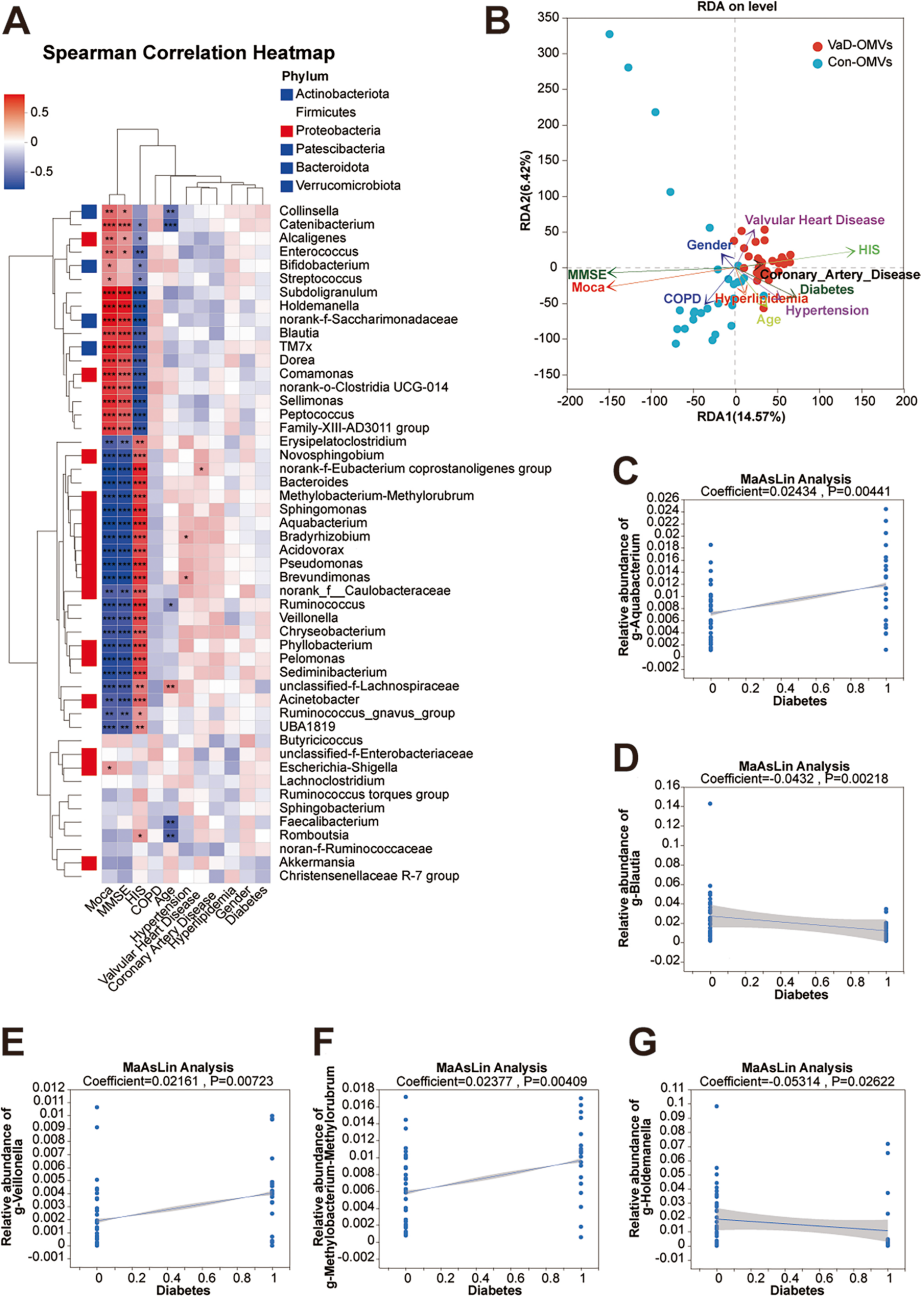
Association between VaD-OMV-derived microbiome and clinical parameters. **A** Heatmap of Spearman correlations between bacterial abundance and clinical factors. **B** Redundancy analysis (RDA) ordination plot showing the relationships between microbial composition and clinical variables. **C–G** MaAsLin2-identified genera significantly associated with diabetes: *Aquabacterium* (**C**), *Blautia* (**D**), *Veillonella* (**E**), *Anaeroglobus* (**F**), and *Methylobacterium-Methylorubrum* (**G**). Abbreviations: VaD-OMVs, vascular dementia-derived outer membrane vesicles; Con-OMVs, control-derived outer membrane vesicles; MaAsLin, multivariate association with linear models

Spearman correlation heatmap analysis was used to identify which OMV-associated FM exhibited relatively strong correlations with clinical factors. The results demonstrated that the MMSE and MoCA scores were highly positively correlated with the *Family-XIII-AD3011 group*, *Peptococcus*, *Sellimonas*, *norank-*o-*Clostridia*-*UCG*-*014*, *Comamonas*, *Dorea*, TM7x, *Blautia*, *norank*-*f*-*Saccharimonadaceae*, *Holdemanella*, and *Subdoligranulum*. Conversely, these clinical scores were highly negatively correlated with *Acinetobacter*, *unclassified*-*f*-*Lachnospiraceae*, *Sediminibacterium*, *Pelomonas*, *Phyllobacterium*, *Chryseobacterium*, *Veillonella*, *Ruminococcus*, *Brevundimonas*, *Pseudomonas*, *Acidovorax*, *Bradyrhizobium*, *Aquabacterium*, *Sphingomonas*, *Methylobacterium*-*Methylorubrum*, *Bacteroides*, *norank*-*f*-*Eubacterium*-*coprostanoligenes*-*group*, and *Novosphingobium* (Fig. 11A). These microorganisms may represent key species influencing VaD-related clinical factors and could thus serve as one approach for screening microbial biomarkers distinguishing the VaD group from the healthy control group.

Clinical information from patients was subsequently collected to investigate the relationships between the microbial abundance of OMVs and clinical factors. Considering the correlations among clinical risk factors, a preliminary screening of the clinical data was performed prior to the clinical risk factor association analysis. The clinical information selected for this study included age, diabetes status, COPD status, etc. RDA/CAA revealed that diabetes exerted the most prominent influence on the FM derived from OMVs (Fig. 11B). Further analysis via MaAsLin revealed that the diabetic status of VaD patients was significantly associated with the enrichment of *Aquabacterium*, *Blautia*,* Veillonella*, *Methylobacterium-Methylorubrum* and *Holdemanella* (all *P* < 0.01) (Fig. 11C-G).

## Discussion

This study reveals for the first time that OMVs derived from the GM of VaD patients can migrate from the intestinal tract into the brain tissue of mice and demonstrates significant alterations in the microbial composition represented by OMVs from VaD patients. In the VaD group, the abundances of potentially pathogenic bacteria such as *Pseudomonas*, *Acinetobacter*, and *Brevundimonas* increased, whereas those of beneficial bacteria, including *Bifidobacterium* and *Faecalibacterium*, significantly decreased. These findings provide a basis for subsequent multiomics analysis and mechanistic investigations and establish a theoretical foundation for developing OMV-based precision delivery systems, regional VaD prevention and control strategies, and localized microbial interventions.

Accumulating evidence in recent years has highlighted the significant role of FM-derived OMVs in the pathogenesis of cerebrovascular diseases, such as stroke and cerebral aneurysms. OMVs can activate immune responses and promote the release of inflammatory factors, which constitute a central mechanism underlying cerebrovascular pathology [21, 22]. Moreover, by triggering inflammatory reactions in cells of the blood‒brain barrier, OMVs increase their permeability, thereby facilitating the entry of pathological substances into the brain parenchyma [23]. Once within the brain, OMVs can further activate immune cells, including microglia, exacerbating neuroinflammatory responses and contributing to vascular injury [24, 25]. The RNA, proteins, and lipids carried by OMVs are capable of modulating neuronal and vascular endothelial functions and may even propagate across different brain regions, amplifying the extent of damage or accelerating disease progression [26–28]. As important mediators of intercellular communication, OMVs are likely to exert pleiotropic effects on the initiation and development of cerebrovascular disorders. Given that VaD is a form of cognitive impairment closely linked to cerebrovascular damage, the composition and functional characteristics of intestinal OMVs in VaD patients remain poorly investigated and merit further in-depth exploration.

In the present study, we employed, for the first time, 16 S rRNA V3–V4 sequencing to analyze the microbiome associated with intestinal OMVs in patients with VaD. Our results demonstrate that OMVs derived from VaD patients can translocate from the gastrointestinal tract into the brain and be widely distributed in mice. Specifically, these OMVs have been detected in multiple brain regions critically involved in cognitive function, including the frontal association cortex (FrA)/secondary motor cortex (M2) [33, 34], cingulate cortex (Cg) [35], lateral part of the accumbens shell (LAcbSh) [36], caudate putamen (CPu) [37], and dentate gyrus (DG)/Cornu Ammonis area 1 (CA1) [38, 39]. Furthermore, OMVs are also present in regions with more indirect or specialized cognitive roles, such as the lateral globus pallidus (LGP) [40], triangular septal nucleus (TS) [41], and anterior olfactory nucleus (AOD/AOV) [42]. The specific localization of OMVs within key nodes of cognitive circuits—including the hippocampus (DG/CA1) for memory [43], frontal cortex for executive function [44, 45], and striatal regions (CPu, AcbSh) for motivation [46, 47] and reward—strongly suggests their potential to directly disrupt the neural processes underlying cognition. These findings support the proposed mechanism whereby FM-derived OMVs may enter the circulation and cross the blood‒brain barrier, thereby contributing to neuropathological processes [48, 49]. While the presence of bacterial components in the bloodstream is well documented, their cellular origin remains debated: some researchers hypothesize gastrointestinal leakage, whereas others suggest derivation from the skin or oral cavity, particularly under conditions of barrier compromise [50–52]. Our results provide experimental evidence that OMVs are a potential source of such components. Overall, we propose that OMVs produced by the disturbed FM in VaD may represent a previously underappreciated mechanism contributing to disease progression via the gut‒brain axis.

α-Diversity analysis revealed that the Ace, Chao, and Sobs indices were lower in the VaD-OMV group than in the control group, which is consistent with several previous studies on the VaD microbiota [17, 18]. This reduction may reflect greater community similarity among samples within the VaD group. Although the Shannon index showed an increasing trend, the difference was not statistically significant, suggesting that further studies with larger sample sizes are needed to confirm this observation.

This study elucidates the assembly principles and core structure of the microbial community within FM-derived OMVs from VaD patients. We identified a strong positive correlation between species prevalence and relative abundance (*R²* = 0.750, *P* < 0.001), indicating that the assembly of the OMV microbial community adheres to the classic ecological “abundance-distribution” law and is nonrandom [53]. Further community structure analysis revealed that while microbial diversity is maintained by many rare and transient taxa, ecological function (measured by total biomass) is highly concentrated in a small number of persistent taxa (accounting for 79.24%-83.8%). These findings suggest that the function of VaD-associated OMVs is driven primarily by a small, core set of persistent species. Crucially, among the identified core keystone taxa, genera such as *Acinetobacter* and *Pseudomonas* were identified as network hubs, and these same taxa were significantly enriched in VaD-OMVs. These findings indicate that these conditionally pathogenic bacteria, which proliferate in the VaD environment, may exert a disproportionate core effect via their OMVs in gut‒brain axis communication; their dynamics could amplify detrimental impacts on the entire community and the host. We conclude that the microbial community in OMVs from VaD patients is dominated by a core keystone taxon enriched with conditionally pathogenic bacteria, providing a novel structural foundation for understanding the specific role of OMVs in the pathological mechanisms of VaD.

We further analyzed the taxonomic origins of OMVs. *Firmicutes* and *Bacteroidota* constitute more than 80% of the FM community [54]. The *Firmicutes*/*Bacteroidetes* (F/B) ratio is crucial for gut homeostasis, and its dysregulation is linked to various diseases [55, 56]. For example, an elevated F/B ratio is associated with diabetes and obesity [57, 58], whereas a decreased ratio is observed in patients with inflammatory bowel disease and nonalcoholic fatty liver disease/steatohepatitis (NAFLD/NASH) [59, 60]. Thus, the F/B ratio may predict inflammation-related changes, as *Firmicutes* exert anti-inflammatory effects that may alleviate IBD progression, whereas Bacteroidota may promote cytokine-driven intestinal inflammation [61]. In this study, VaD patients presented a reduced abundance of *Firmicutes* and increased abundance of Bacteroidota, resulting in a lower F/B ratio, suggesting its potential as a biomarker for VaD activity. However, owing to the limited sample size and heterogeneous treatment responses, it remains unclear whether the F/B ratio changes stem from pharmacological intervention or disease remission. Although confounding factors were partially controlled, dietary and medication variables may still influence the results. Limitations in species-level resolution and functional profiling also warrant consideration.

At the genus level, VaD patients presented increased abundances of *Pseudomonas*, *Acinetobacter*, and *Brevundimonas*, along with a decrease in beneficial genera such as *Bifidobacterium* and *Faecalibacterium*, compared with those in the control group. *Pseudomonas* enrichment has been associated with cognitive impairment, potentially via amyloid production stimulation [62], and may affect the central nervous system through gut–brain axis metabolites [63]. *Acinetobacter*, an opportunistic pathogen, can trigger infections in immunocompromised hosts [64]. These findings suggest that these bacterial groups not only are “bystanders” in the gut microenvironment but also may actively contribute to neuroinflammation and cognitive impairment in VaD.

The reduction in *Faecalibacterium abundance* in VaD patients is notable, as its depletion is also observed in IBD, diabetes, and chronic kidney disease [65–67]. Takuji et al. compared the FM among healthy individuals, those with mild cognitive impairment (MCI), and Alzheimer’s disease (AD) patients and identified *Faecalibacterium* as a potentially beneficial genus for preventing MCI. In an Aβ-injected mouse model, strains Fp14 and Fp360 of *Faecalibacterium* were shown to improve cognitive function, potentially via the mitigation of cerebral oxidative stress and the regulation of mitochondrial function [67].

Moreover, this study revealed a marked decrease in the abundance of *Bifidobacterium* within OMVs from VaD patients compared with controls. Clinical studies indicate that Bifidobacterium supplementation improves neurological function, cognitive performance, and immune regulation in elderly stroke patients, as demonstrated by improved NIHSS and MoCA scores alongside FM alterations [68]. The mechanism may involve enhanced intestinal barrier function, increased short-chain fatty acid production, and antioxidant/antineuroinflammatory effects mediated by *Bifidobacterium*-derived OMVs [69]. Animal studies have confirmed that *Bifidobacterium BGN4* promotes neuronal regeneration in aging models [70], whereas its immunomodulatory role, characterized by reduced proinflammatory cytokines (IL-6, IL-1β, TNF-α) and elevated immunoglobulins, is particularly relevant to VaD pathophysiology [68]. In summary, Bifidobacterium has significant cognitive regulatory functions, suggesting that its supplementation or OMV-based delivery represents a promising microbe-targeted strategy for VaD treatment.

In the diagnostic model, OMVs derived from *Holdemanella* had the highest feature importance (contribution value = 6.2427). As an important gut commensal bacterium, Holdemanella produces metabolites that activate intestinal L cells to secrete GLP-1, thereby improving glucose metabolism and enhancing neural signaling [71–73]. We speculate that OMVs may serve as key mediators linking metabolic disorders to cerebrovascular impairment and hold potential as probiotic candidates for VaD intervention. Moreover, the significantly reduced abundance of Bifidobacterium in the OMVs of VaD patients further underscores the crucial role of protective microbiome loss in cognitive impairment.

Correlation analysis revealed that the MMSE and MoCA scores were significantly associated with several OMV-related microorganisms. For example, genera such as Holdemanella and Sabacter are positively correlated with cognitive function, potentially exerting neuroprotective effects through mechanisms such as short-chain fatty acid production, maintenance of intestinal barrier integrity, and immune regulation. In contrast, conditional pathogens such as *Acinetobacter*, *Pseudomonas*, and *Brevundimonas* were negatively correlated with cognitive scores. Their enrichment may exacerbate systemic neuroinflammation via pathogen-associated molecular patterns (PAMPs) carried by OMVs, disrupting the blood‒brain barrier and promoting VaD progression.

In summary, this study establishes the value of diabetes-related OMV microbial markers in VaD diagnosis, with *Holdemanella* and *Bifidobacterium* serving as potential positive and negative regulators, respectively, together forming key targets for microbiome-based intervention in VaD. Future research should focus on validating the specific mechanisms of these key OMVs and exploring their applications in precise diagnosis and targeted therapy. Furthermore, in functional mechanism exploration, we identified differences in microbial signatures between VaD-OMVs and Con-OMVs through microbiome data analysis and predicted their associations with amino acid metabolism pathways. Subsequent metabolomic analysis was conducted to infer how VaD-OMVs may influence VaD progression via biological processes related to amino acid metabolism. While previous studies have demonstrated that bacterial OMVs can cross epithelial barriers and enter the circulation while maintaining their cargo and functional activity [74, 75], we acknowledge that without additional experimental validation, such as co-localization studies using OMV-specific protein markers, genetically engineered fluorescent protein-labeled OMVs, or transmission electron microscopy of brain-isolated OMVs, we cannot definitively conclude that the observed fluorescence represents exclusively intact OMVs with preserved structural integrity.

This study is positioned at the forefront of international FM research and presents the first high-throughput analysis of FM-derived OMVs in a VaD population from a tropical region of China. A series of differentially abundant bacteria were identified, and a diagnostically promising model was constructed. However, several limitations should be cautiously considered, informing future research directions. While fecal microbiome provides valuable insights into microbial composition, it primarily reflects luminal contents and may not fully represent mucosa-adherent or region-specific microbial communities, which could play distinct roles in host-microbe interactions and disease pathogenesis [76]. Future studies incorporating mucosal biopsies or region-specific sampling along the gastrointestinal tract would complement our findings and provide a more comprehensive view of the microbiome-OMV-host axis in VaD.

Although metabolic comorbidities were adjusted for in our multivariable regression models, stratified analyses were precluded by limited subgroup sample sizes. We cannot entirely exclude the possibility that some microbial alterations may interact with or be partially driven by metabolic dysregulation, particularly given the established links among diabetes, hyperlipidemia, and cognitive impairment. Additionally, commonly used medications for these conditions (e.g., statins, metformin) are known to influence gut microbial composition, and detailed medication records were unavailable for further adjustment.

At the molecular level, while integrated multiomics analyses suggest potential roles of genera such as *Holdemanella* in VaD-OMVs, the specific mechanisms through which key metabolites (e.g., 3-hydroxyoctadecenoic acid) cross the blood‒brain barrier and act on VaD remain unclear. Future investigations employing spatial metabolomics (DESI-MSI), organoid and blood‒brain barrier chip coculture models, and CRISPRi metabolic pathway modulation will help validate the causality and targets of their neuroprotective effects. The core innovation of this study lies in the use of OMVs as biomarker carriers, providing a new perspective to overcome the “black box” limitations of traditional microbiome research.

## Conclusion

Our findings establish OMVs as a critical conduit in the gut-brain axis of VaD, linking intestinal microbial dysbiosis to central neurological pathology. This study identifies and functionally characterizes a distinct OMV-associated microbial signature in VaD patients, marked by the enrichment of conditional pathogens and the depletion of beneficial taxa. The integration of high-throughput sequencing, machine learning, and functional prediction supports a disease model centred on OMV-mediated dissemination of pro-inflammatory and metabolic effectors to the brain. A random forest model derived from this OMV signature demonstrates promising diagnostic potential, highlighting its translational value for early detection and risk stratification. With further validation, this OMV-centric framework may advance the development of precision diagnostics and microbiome-targeted therapies for VaD.

## Supplementary Information

Supplementary Material 1.

Supplementary Material 2.

Supplementary Material 3.

## Data Availability

The data supporting the conclusions of this study have been publicly stored in Figshare, and the website address is 10.6084/m9.figshare.31066648. The raw 16 S rRNA gene sequencing datasets generated and analysed during the current study are available in the NCBI Sequence Read Archive (SRA) under BioProject ID PRJNA1444325 (SRA Run accessions: SRR37824738–SRR37824794). The dataset can be accessed directly at: https://www.ncbi.nlm.nih.gov/bioproject/PRJNA1444325.

## Abbreviations

Ace: Abundance-based Coverage Estimator
AD: Alzheimer’s Disease
AOD: Anterior Olfactory nucleus, dorsal part
AOV: Anterior Olfactory nucleus, ventral part
ASV: Amplicon Sequence Variant
AUC: Area Under the Curve
CA1: Cornu Ammonis area 1
Cgl: Cingulate cortex, lateral part
CI: Confidence Interval
COG: Clusters of Orthologous Groups
Con-OMVs: Control-derived Outer Membrane Vesicles
COPD: Chronic Obstructive Pulmonary Disease
CPHZ: Central People’s Hospital of Zhanjiang
CPu: Caudate Putamen
DG: Dentate Gyrus
F/B ratio: Firmicutes/Bacteroidetes ratio
FDR: False Discovery Rate
FrA: Frontal Association cortex
FM: Fecal microbiome
GMHI: Gut Microbiome Health Index
HIS: Hachinski Ischemic Scale
IBD: Inflammatory Bowel Disease
KEGG: Kyoto Encyclopedia of Genes and Genomes
LAcbSh: lateral part of the Accumbens Shell
LDA: Linear Discriminant Analysis
LEfSe: Linear Discriminant Analysis Effect Size
LGP: Lateral Globus Pallidus
MaAsLin: Multivariate Association with Linear Models
MCI: Mild Cognitive Impairment
MDI: Microbial Dysbiosis Index
M2: secondary Motor cortex
MMSE: Mini-Mental State Examination
MoCA: Montreal Cognitive Assessment
Mol: Molecular layer of the cerebellum
NAFLD: Nonalcoholic Fatty Liver Disease
NASH: Nonalcoholic Steatohepatitis
NTA: Nanoparticle Tracking Analysis
OMVs: Outer Membrane Vesicles
OTU: Operational Taxonomic Unit
PAMPs: Pathogen-Associated Molecular Patterns
PCoA: Principal Coordinate Analysis
PH: Posterior Hypothalamic area
PICRUSt: Phylogenetic Investigation of Communities by Reconstruction of Unobserved States
PLS-DA: Partial Least Squares-Discriminant Analysis
RDA: Redundancy Analysis
ROC: Receiver Operating Characteristic
SPF: Specific Pathogen-Free
TEM: Transmission Electron Microscopy
TS: Triangular Septal nucleus
VaD: Vascular Dementia
VaD-OMVs: Vascular Dementia-derived Outer Membrane Vesicles
