# Correction: Altered microbial cargo in fecal microbiome-derived outer membrane vesicles as novel biomarkers for vascular dementia

**DOI:** 10.1186/s12866-026-05413-w

**Published:** 2026-07-18

**Authors:** Xin Li, Wei Wei, Shouchao Wei, Wenwei Xu, Lang Mo, Junjun Wang, He Zhu, Zhou Liu, Fengri Jin

**Affiliations:** 1 Zhanjiang Institute of Clinical Medicine, Central People’s Hospital of Zhanjiang, Yuanzhu Road, Zhanjiang, Guangdong China; 2 Medical Center, Gaomi People’s Hospital, Zhenfu Street, Weifang, Shandong China; 3 The Third Department of Neurology, Central People’s Hospital of Zhanjiang, Yuanzhu Road, Zhanjiang, Guangdong China; 4 Office of Drug Clinical Trial Institution, Central People’s Hospital of Zhanjiang, Yuanzhu Road, Zhanjiang, Guangdong China; 5 Department of Physiology, School of Basic Medical Sciences, Guangdong Medical University, Renmindadao Street, Zhanjiang, Guangdong China; 6 Institute of Neurology, The Affiliated Hospital of Guangdong Medical University, Zhanjiang, Guangdong China

**Correction: BMC Microbiol 26**,** 525 (2026).**

**https://doi.org/10.1186/s12866-026-05040-5.**

Following publication of the original article [1], The author reported that in the section ‘Fluorescent Labeling of OMVs’ under ‘Materials and Methods,’ the term PKH26 should be PKH67.

The original article has been updated.
