# Supplementary material for: Altered microbial cargo in fecal microbiome-derived outer membrane vesicles as novel biomarkers for vascular dementia
Source: BMC Microbiol. 2026 Apr 21;26:525. doi: 10.1186/s12866-026-05040-5 (PMC13235108; doi:10.1186/s12866-026-05040-5)
Supplement: Supplementary file 1 — Supplementary Material 1. [file 12866_2026_5040_MOESM1_ESM.docx]

**Supplementary Materials**

**Supplementary Methods**

**Patients and samples collection**

**Inclusion criteria for the vascular dementia (VaD) group**

(1) Diagnosis of vascular dementia (VaD) confirmed by the following clinical criteria: Diagnostic and Statistical Manual of Mental Disorders, Fourth Edition, Revised (DSM-IV-R); International Classification of Diseases, 10th Revision (ICD-10);National Institute of Neurological Disorders and Stroke-Association Internationale pour la Recherche et l’Enseignement en Neurosciences (NINDS-AIREN) criteria; *Chinese Diagnostic Guidelines for Vascular Cognitive Impairment (2017)* issued by the Stroke Prevention and Control Engineering Committee of the National Health and Family Planning Commission of China.

(2) Hachinski Ischemic Scale score ≥7.

(3) Neuropsychological scale assessments at CPHZ confirmed cognitive impairment, with Mini-Mental State Examination (MMSE) scores meeting the following education-adjusted cutoffs: Illiterate: ≤17; Primary education: ≤20; Secondary education: ≤22; Tertiary education: ≤23.

(4) Cerebral infarction caused by large artery atherosclerotic thrombosis, as classified by the modified TOAST criteria.

**Inclusion criteria for control group**

(1) No significant subjective complaints of memory decline or focal neurological symptoms;

(2) Clinical Dementia Rating (CDR) score of 0, normal global cognitive function assessment, Mini-Mental State Examination (MMSE) scores exceeding the cutoff values for corresponding educational levels.

**Exclusion criteria (both case and control groups)**

(1) Diagnosed with dementia, depression, or other psychiatric disorders due to non-vascular etiologies according to the *Diagnostic and Statistical Manual of Mental Disorders, Fourth Edition, Revised* (DSM-IV-R) criteria;

(2) Cognitive impairment caused by severe traumatic brain injury, epilepsy, Parkinson’s disease, or related conditions;

(3) Severe systemic diseases affecting other organ systems;

(4) History of antibiotic use within the preceding 3 months;

(5) Severe substance abuse (e.g., tobacco, alcohol);

(6) Chronic intestinal diseases;

(7) Respiratory system diseases.

**Sample Size Estimation and Statistical Methods**

Given the high interindividual variability of gut microbiota and the absence of standardized sample size guidelines for gut microbiome-derived OMVs studies, our sample size determination referenced prior high-throughput sequencing investigations (typically 20–30 subjects per group) (1-3). After comprehensive review of literature and team discussions balancing scientific rigor, resource availability, and feasibility, we recruited 29 VaD patients and 28 controls. Clinical data were analyzed using SPSS 20.0 (IBM Corp.). Continuous variables are expressed as mean ± standard deviation (SD). Normality was assessed using Shapiro-Wilk tests. Parametric (independent t-test) or nonparametric (Mann-Whitney U test) methods were applied for intergroup comparisons of continuous variables, while categorical variables were analyzed using χ² or Fisher’s exact tests. A two-tailed statistical significance threshold (α = 0.05) was adopted.

**Experimental mice**

The experimental animals used in this study were two-week-old male C57BL/6J mice (specific pathogen-free [SPF] grade, body weight 20 ± 3 g), provided by Hangzhou Ziyuan Biotechnology Co., Ltd. (License No.: SCXK [Zhe] 2019-0004). Adaptive housing was conducted at the SPF-level animal experimental center of Guangdong Medical University. All animal experimental protocols and procedures were reviewed and approved by the Institutional Animal Ethics Committee of Guangdong Medical University. The animal facility environment was precisely regulated by a bioclean system: a circadian rhythm simulation system maintained a 12/12-hour light/dark cycle (light intensity 15–20 lux), temperature fluctuations within 20–24°C (gradient ≤0.5°C/h), and relative humidity of 60%–70% (±5% deviation). The experimental animals had ad libitum access to gamma-irradiated sterilized standard rodent diet and autoclaved triple-purified water.

**Clarification on Animal Anesthesia and Euthanasia Methods**

All animal experimental procedures in this study were conducted in strict compliance with the *Implementation Rules for the Management of Laboratory Animals at Guangdong Medical University* (https://sydwzx.gdmu.edu.cn/info/1006/1077.htm) and the internationally recognized "3Rs" principles of animal welfare (Replacement, Reduction, and Refinement). The protocols were reviewed and approved by the Institutional Animal Ethics Committee of Guangdong Medical University (Approval No. GDY2403466).

**Anesthesia Method**

(1) Purpose: To ensure animals were in a state of deep analgesia and unconsciousness prior to brain tissue perfusion and collection, thereby eliminating pain and stress during surgical procedures.

(2) Anesthetic State: Experimental animals were all subjected to deep anesthesia prior to euthanasia, ensuring they remained completely unconscious throughout the critical operative steps.

(3) Anesthetic Agent: 1.25% tribromoethanol (Avertin) solution (M2920, Nanjing AlBi Bio-Technology Co..Ltd, China) was used. This agent is a commonly used short-acting anesthetic characterized by rapid onset, moderate duration of anesthesia, and stable anesthetic depth, making it suitable for acute surgical procedures in rodent models.

(4) Dosage and Route of Administration: M2920 is a ready-to-use sterile solution containing 1.25% (v/v) tribromoethanol (Avertin), tertiary amyl alcohol, and 0.9% physiological saline, with a final tribromoethanol concentration of 20 mg/ml. Administration was via intraperitoneal injection at a dose of 0.2 ml/10 g body weight for mice. This dosage ensures that animals enter a stable surgical plane of anesthesia within minutes, as indicated by the loss of corneal and toe-pinch reflexes.

**Euthanasia/Sacrifice Method**

Following deep anesthesia (unconsciousness), transcardiac perfusion fixation was employed as the terminal procedure. This method enables both humane euthanasia and optimal preservation of tissue architecture for high-quality neurohistological analysis. Detailed Procedure:

(1) The deeply anesthetized mouse was positioned supine and secured on a surgical board.

(2) The thoracic cavity was quickly opened to expose the heart.

(3) A perfusion needle was inserted into the ascending aorta via the left ventricle, and the right atrial appendage was incised to serve as an outflow tract.

(4) Approximately 20 mL of ice-cold 0.9% physiological saline was rapidly perfused first, until the liver and lungs turned pale and the effluent from the right atrial appendage ran clear, ensuring thorough removal of blood from the vasculature.

(5) This was followed by perfusion with approximately 50 mL of ice-cold 4% paraformaldehyde (PFA) in phosphate buffer, continuing until rigidity and tremors were observed in the limbs, tail, and torso of the animal, after which perfusion was stopped.

**Rationale for Selection**

(1) Humane Endpoint: At the initiation of perfusion, the animal was already in a deeply anesthetized, unconscious state. The perfusion process itself does not induce any sensation of pain or awareness.

(2) Scientific Necessity: Transcardiac perfusion fixation instantaneously halts cellular metabolism and maximally preserves the native morphological structure and antigenicity of brain tissue. This is crucial for subsequent immunofluorescence staining and precise observation of the distribution of OMVs within the brain. Compared to methods such as cervical dislocation or anesthetic overdose alone, this approach provides optimal quality assurance for histological outcomes.

(3) Regulatory Compliance: This method aligns with the *American Veterinary Medical Association* (AVMA) Guidelines for the Euthanasia of Animals (https://www.avma.org/), which recommend certain physical methods for animals that are already anesthetized. In summary, the design of the animal anesthesia and euthanasia protocol in this study prioritized animal welfare, completely avoiding unnecessary suffering. Concurrently, it fulfilled the high technical requirements of the experiment for brain tissue morphological integrity, thereby providing a methodological foundation for the reliability of the research findings.

**References**

1. Kang S, Kang J, Park G-S, Ko S-H, Jang Y, Park J-H, et al. Comparison of mucosal microbiomes from biliary tract among patients with extrahepatic cholangiocarcinoma, patients with chronic cholecystitis, and healthy controls. 2024;42(3_suppl):550-.

2. Jansen VL, Davids M, van Mourik DJ, Levels JH, Coppens M, Middeldorp S, et al. Gut microbiome composition and intestinal immunity in antiphospholipid syndrome patients versus healthy controls. Lupus. 2024;33(12):1373-8.

3. Xu ZJ, Chen L, Tang QL, Li D, He CJ, Xu CL, et al. Differential oral and gut microbial structure related to systemic metabolism in kidney stone patients. World journal of urology. 2024;42(1):6.

**Supplementary Figures**


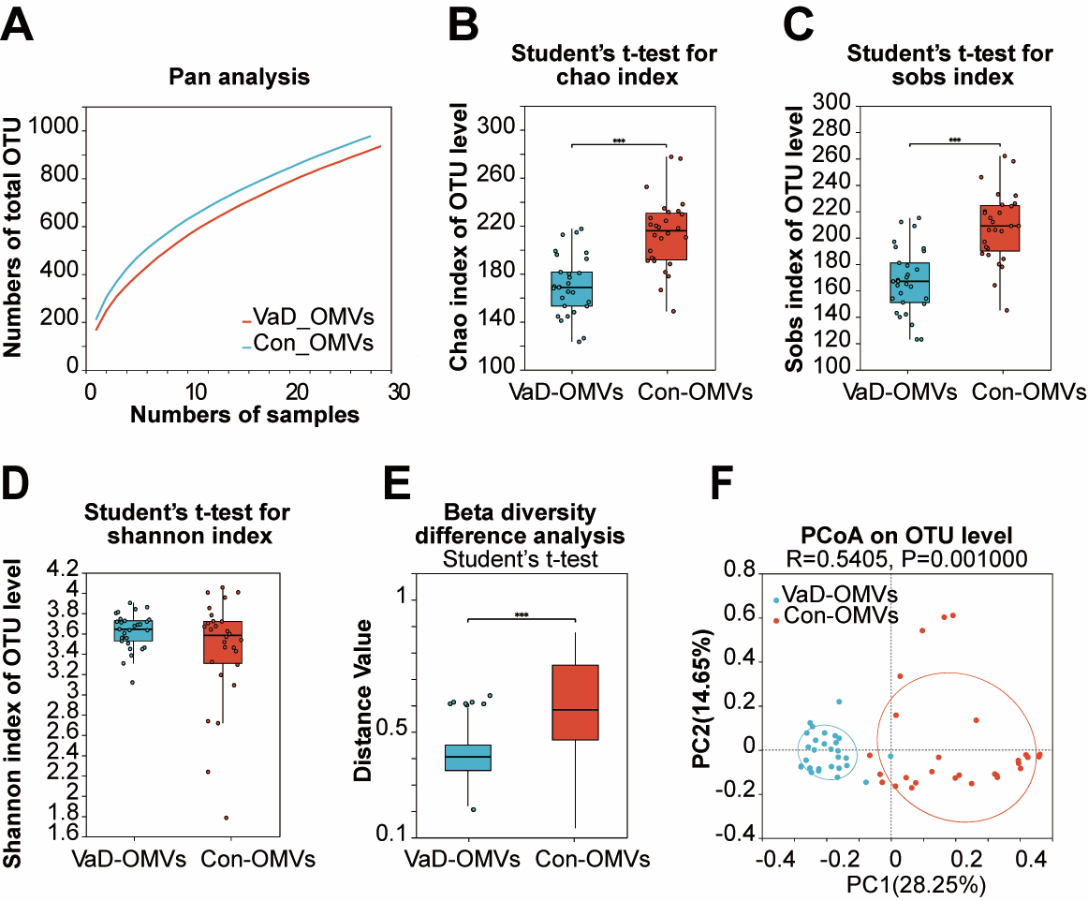


**Supplemental Fig. 1 Microbial diversity and dysbiosis analysis of VaD-OMV-associated microbiota.** (**A**) Pan species curves representing total species richness. (**B-D**) α diversity indices at the OTU level: Chao (**B**), Sobs (**C**) and Shannon (**D**). (**E–F**) β diversity analyses: intergroup dissimilarity (**E**) and PLS-DA (**F**). Abbreviations: VaD-OMVs, vascular dementia-derived outer membrane vesicles; Con-OMVs, control-derived outer membrane vesicles; OTU, operational taxonomic unit; GMHI, Gut Microbiome Health Index; MDI, Microbial Dysbiosis Index; PCoA, principal coordinate analysis; PLS-DA, partial least squares-discriminant analysis; OMVs, outer membrane vesicles.
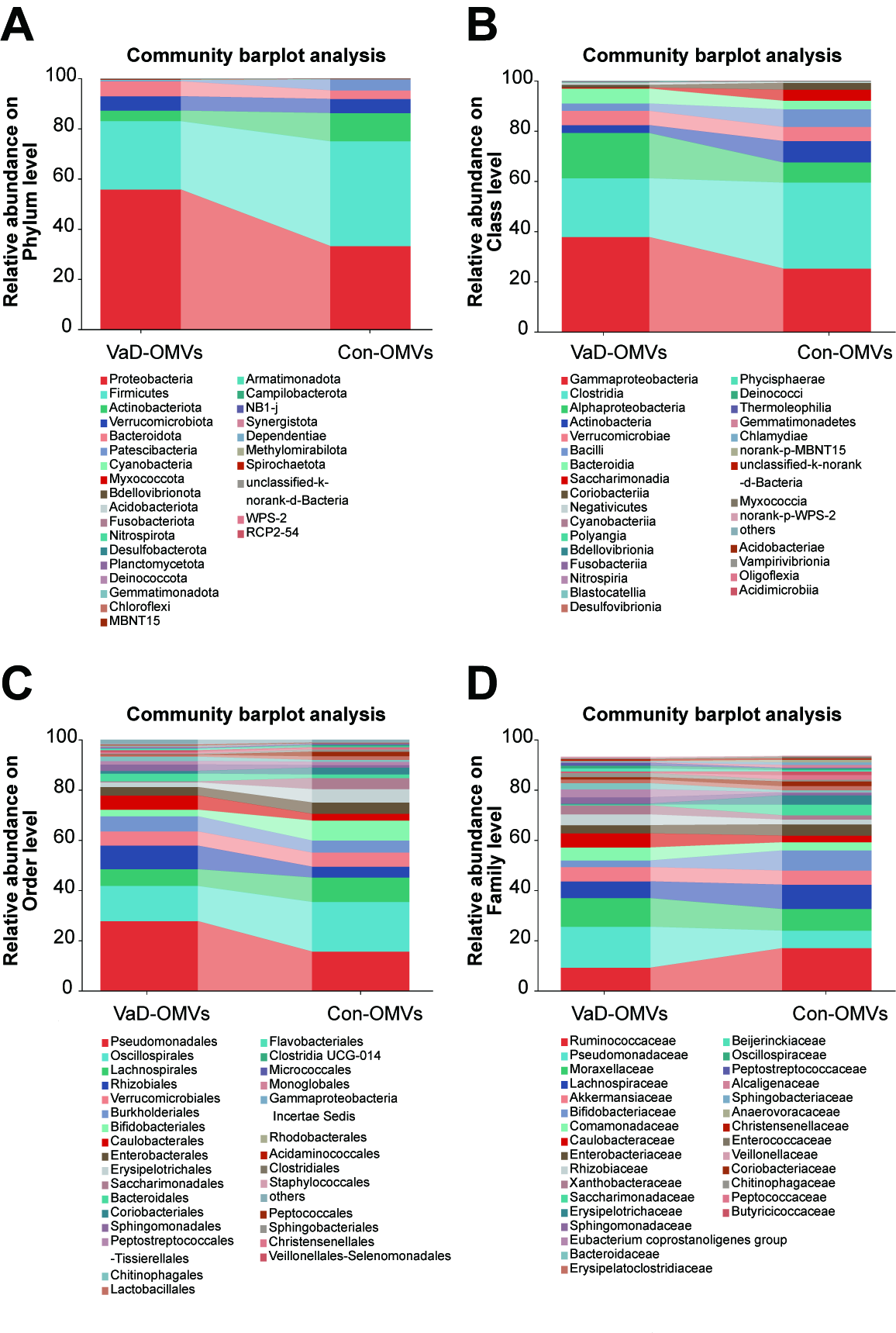


**Supplemental Fig. 2 Relative abundance of GM communities at the phylum.** (A), class (B), order (C) and family (D) levels. **Abbreviations**: VaD-OMVs, vascular dementia-derived outer membrane vesicles; Con-OMVs, control-derived outer membrane vesicles; GM, Gut Microbiota.

**Supplementary Table**

**Supplementary Table 1** Comparison of microbial taxonomic abundance between the VaD-OMVs group and the control group (mean ± SD)

| Level | Taxon (Latin Name) | VaD-OMVs | Con-OMVs | Trend | P-value |
| --- | --- | --- | --- | --- | --- |
| Phylum | Proteobacteria | 55.73±9.833 | 33.18±18.57 | ↑ | <0.05 |
|  | Bacteroidota | 5.97±2.11 | 3.41±2.85 | ↑ | <0.05 |
|  | Firmicutes | 27.22±8.82 | 41.74±18.27 | ↓ | <0.05 |
|  | Actinobacteriota | 4.215±2.567 | 11.26±8.896 | ↓ | <0.05 |
|  | Patescibacteria | 0.40±0.39 | 4.39±3.35 | ↓ | <0.05 |
| Class | Gammaproteobacteria | 37.72±9.20 | 25.15±16.54 | ↑ | <0.05 |
|  | Alphaproteobacteria | 18.01±3.58 | 8.02±5.371 | ↑ | <0.05 |
|  | Bacteroidia | 5.97±2.11 | 3.41±2.85 | ↑ | <0.05 |
|  | Clostridia | 23.45±8.032 | 34.27±19.24 | ↓ | <0.05 |
|  | Actinobacteria | 3.13±2.49 | 8.51±7.20 | ↓ | <0.05 |
|  | Saccharimonadia | 0.39±0.39 | 4.38±3.35 | ↓ | <0.05 |
| Order | Pseudomonadales | 27.74±5.692 | 15.6±10.75 | ↑ | <0.05 |
|  | Rhizobiales | 9.37±2.061 | 4.30±3.1 | ↑ | <0.05 |
|  | Burkholderiales | 6.02±1.45 | 4.8±4.20 | ↑ | <0.05 |
|  | Caulobacterales | 5.65±1.36 | 2.65±1.70 | ↑ | <0.05 |
|  | Bifidobacteriales | 2.64±2.47 | 8.00±7.28 | ↓ | <0.05 |
|  | Erysipelotrichales | 1.87±1.11 | 5.35±3.17 | ↓ | <0.05 |
|  | Saccharimonadales | 0.39±0.39 | 4.38±3.35 | ↓ | <0.05 |
| Family | Pseudomonadaceae | 16.34±3.69 | 7.01±4.88 | ↑ | <0.05 |
|  | Moraxellaceae | 11.4±4.87 | 8.60±9.25 | ↑ | <0.05 |
|  | Caulobacteraceae | 5.59±1.33 | 2.63±1.67 | ↑ | <0.05 |
|  | Ruminococcaceae | 9.17±5.37 | 16.95±15.90 | ↓ | <0.05 |
|  | Bifidobacteriaceae | 2.64±2.47 | 8.00±7.28 | ↓ | <0.05 |
| Genus | Pseudomonas | 16.34±3.69 | 7.01±4.88 | ↑ | <0.05 |
|  | Acinetobacter | 11.26±4.87 | 8.54±9.24 | ↑ | <0.05 |
|  | Brevundimonas | 4.52±1.19 | 2.05±1.27 | ↑ | <0.05 |
|  | Bifidobacterium | 2.64±2.47 | 8.00±7.28 | ↓ | <0.05 |
|  | Faecalibacterium | 2.61±2.87 | 7.17±17.03 | ↓ | <0.05 |
|  | Subdoligranulum | 1.61±1.85 | 7.29±4.07 | ↓ | <0.05 |
|  | Blautia | 1.17±0.97 | 3.11±2.51 | ↓ | <0.05 |

**Note**: "↑" indicates that the abundance in the VaD-OMVs group was significantly higher than that in the Con-OMVs group, while "↓" indicates it was significantly lower than that in the Con-OMVs group; entries marked with "significant increase" at the genus level did not provide specific numerical values; all comparisons were statistically significant (P < 0.05). **Abbreviations**: VaD-OMVs, vascular dementia-derived outer membrane vesicles; Con-OMVs, control-derived outer membrane vesicles.

**Supplementary Questionnaire**

**Clinical and Cognitive Assessment Questionnaire for Vascular Dementia Study**

Questionnaire ID: __________________

Date of Assessment: ____/____/____

**Part 1: Basic Information and Informed Consent**

1. The participant has been fully informed about the purpose, procedures, and potential risks of this study and voluntarily agrees to participate. I consent to provide relevant information and biological samples for scientific research.

□ Yes

□ No (If No, terminate the assessment)

Signature: __________________ (Participant/Legal Representative) Date: ________

**Part 2: Demographic Information**

2. Age: ______ years

3. Sex:

□ Male

□ Female

**Part 3: Medical History and Comorbidities**

(Please complete based on medical records or reports from the participant/caregiver. Select "Yes" for a confirmed diagnosis.)

4. Hypertension:

□ Yes

□ No

5. Diabetes Mellitus:

□ Yes

□ No

6. Hyperlipidemia:

□ Yes

□ No

7. Valvular Heart Disease:

□ Yes

□ No

8. Chronic Obstructive Pulmonary Disease (COPD):

□ Yes

□ No

**Part 4: Neuropsychological Assessments**

(This section must be completed by a trained assessor following a facetoface interview with the participant.)

A. MiniMental State Examination (MMSE)

Score: ______ / 30

Assessor: _____________ Date of Assessment: ________

B. Montreal Cognitive Assessment (MoCA)

Score: ______ / 30

Assessor: _____________ Date of Assessment: ________

C. Hachinski Ischemic Scale (HIS)

Score: ______

Assessor: _____________ Date of Assessment: ________

**Part 5: Assessor's Notes**

(For recording observations during assessment, special circumstances, or any other relevant remarks)

________________________________________________________________

________________________________________________________________

________________________________________________________________

Assessor's Signature: __________________

Reviewer's Signature: __________________

**Instructions for Use:**

This questionnaire is a researchspecific instrument. Assessors must be trained in the standardized administration of the included scales.

Scoring for the MMSE, MoCA, and HIS must strictly adhere to their official manuals.

All personal information will be anonymized and kept strictly confidential.
