# Supplementary material for: Altered microbial cargo in fecal microbiome-derived outer membrane vesicles as novel biomarkers for vascular dementia
Source: BMC Microbiol. 2026 Apr 21;26:525. doi: 10.1186/s12866-026-05040-5 (PMC13235108; doi:10.1186/s12866-026-05040-5)
Supplement: Supplementary file 2 — Supplementary Material 2. [file 12866_2026_5040_MOESM2_ESM.docx]

**Clinical and Cognitive Assessment Questionnaire for Vascular Dementia Study**

Questionnaire ID: __________________

Date of Assessment: ____/____/____

**Part 1: Basic Information and Informed Consent**

1. The participant has been fully informed about the purpose, procedures, and potential risks of this study and voluntarily agrees to participate. I consent to provide relevant information and biological samples for scientific research.

□ Yes

□ No (If No, terminate the assessment)

Signature: __________________ (Participant/Legal Representative) Date: ________

**Part 2: Demographic Information**

2. Age: ______ years

3. Sex:

□ Male

□ Female

**Part 3: Medical History and Comorbidities**

(Please complete based on medical records or reports from the participant/caregiver. Select "Yes" for a confirmed diagnosis.)

4. Hypertension:

□ Yes

□ No

5. Diabetes Mellitus:

□ Yes

□ No

6. Hyperlipidemia:

□ Yes

□ No

7. Valvular Heart Disease:

□ Yes

□ No

8. Chronic Obstructive Pulmonary Disease (COPD):

□ Yes

□ No

**Part 4: Neuropsychological Assessments**

(This section must be completed by a trained assessor following a facetoface interview with the participant.)

A. MiniMental State Examination (MMSE)

Score: ______ / 30

Assessor: _____________ Date of Assessment: ________

B. Montreal Cognitive Assessment (MoCA)

Score: ______ / 30

Assessor: _____________ Date of Assessment: ________

C. Hachinski Ischemic Scale (HIS)

Score: ______

Assessor: _____________ Date of Assessment: ________

**Part 5: Assessor's Notes**

(For recording observations during assessment, special circumstances, or any other relevant remarks)

________________________________________________________________

________________________________________________________________

________________________________________________________________

Assessor's Signature: __________________

Reviewer's Signature: __________________

**Instructions for Use:**

This questionnaire is a researchspecific instrument. Assessors must be trained in the standardized administration of the included scales.

Scoring for the MMSE, MoCA, and HIS must strictly adhere to their official manuals.

All personal information will be anonymized and kept strictly confidential.
