# Supplementary figures and images for: Altered microbial cargo in fecal microbiome-derived outer membrane vesicles as novel biomarkers for vascular dementia
Source: BMC Microbiol. 2026 Apr 21;26:525. doi: 10.1186/s12866-026-05040-5 (PMC13235108; doi:10.1186/s12866-026-05040-5)

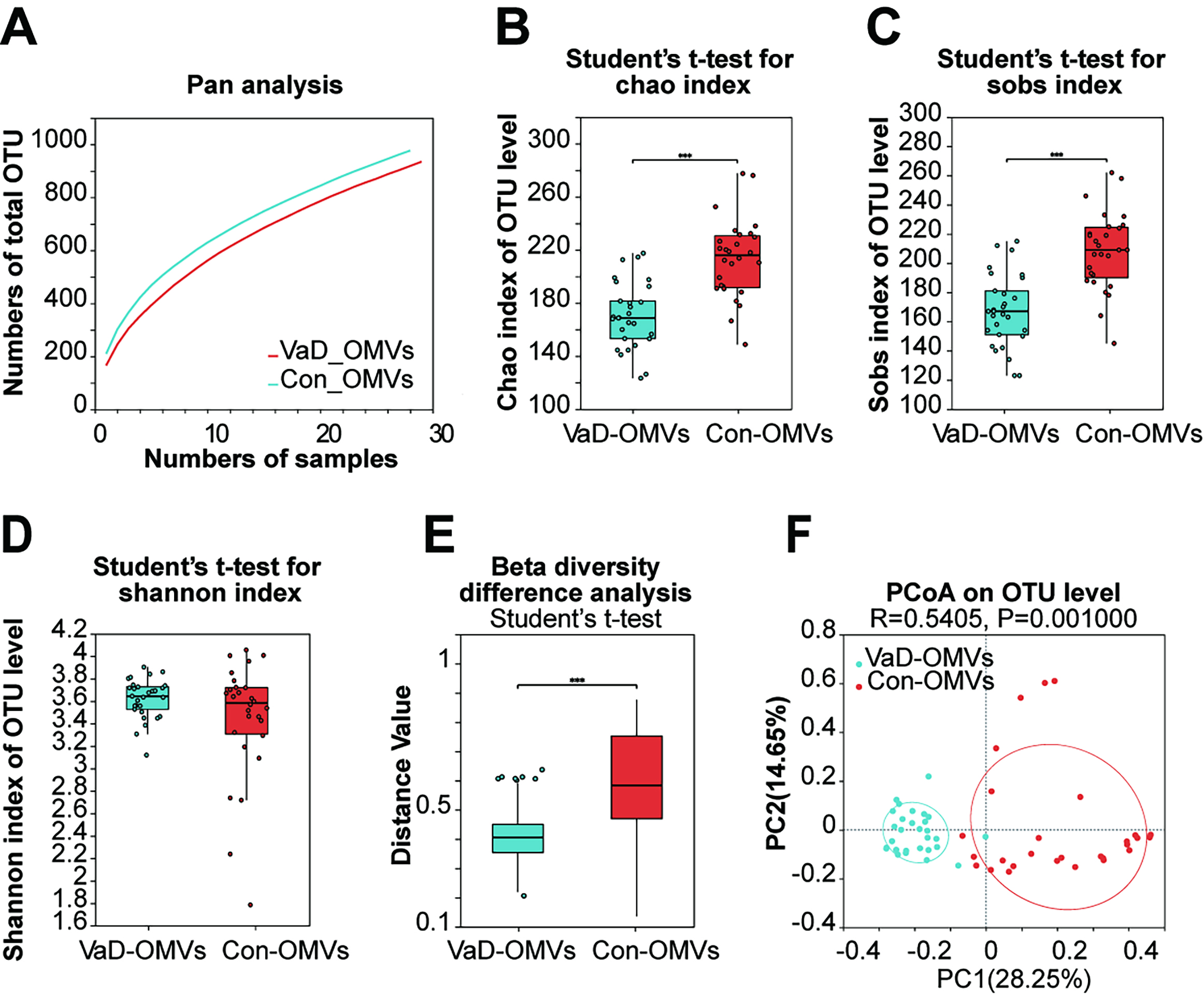

Supplement: Supplementary file 3 — Supplementary Material 3. [file 12866_2026_5040_MOESM3_ESM.zip › Sup Figure_1.jpg]

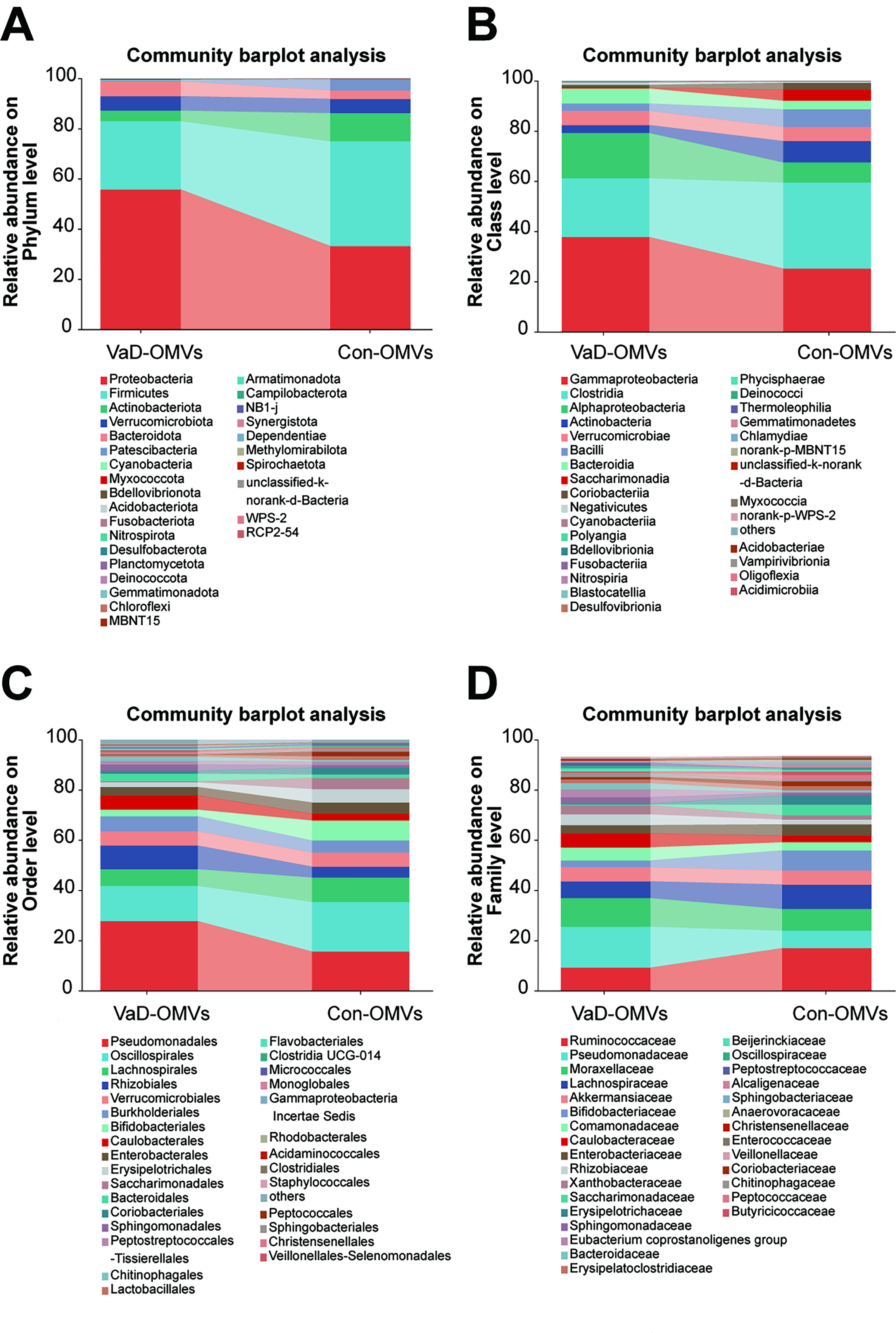

Supplement: Supplementary file 3 — Supplementary Material 3. [file 12866_2026_5040_MOESM3_ESM.zip › Sup Figure_2.jpg]
